# Integrated Treatment of Per- and Polyfluoroalkyl Substances in Existing Wastewater Treatment Plants—Scoping the Potential of Foam Partitioning

**DOI:** 10.1021/acsestengg.3c00091

**Published:** 2023-08-01

**Authors:** Sanne J. Smith, Chantal Keane, Lutz Ahrens, Karin Wiberg

**Affiliations:** † Department of Aquatic Sciences and Assessment, Swedish University of Agricultural Sciences (SLU), P.O. Box 7050, SE-750 07 Uppsala, Sweden; ‡ Queensland Alliance for Environmental Health Sciences, University of Queensland, Woolloongabba, QLD 4102, Australia

**Keywords:** per- and polyfluoroalkyl substances, wastewater treatment, foaming, activated sludge, moving bed biofilm reactors

## Abstract

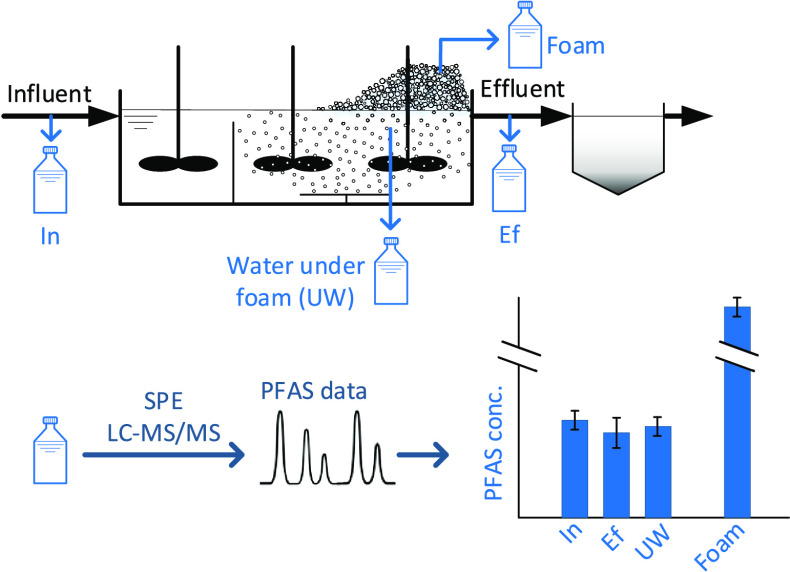

Foam fractionation is becoming increasingly popular as a treatment technology for water contaminated with per- and polyfluoroalkyl substances (PFAS). At many existing wastewater treatment facilities, particularly in aerated treatment steps, foam formation is frequently observed. This study aimed to investigate if foam fractionation for the removal of PFAS could be integrated with such existing treatment processes. Influent, effluent, water under the foam, and foam were sampled from ten different wastewater treatment facilities where foam formation was observed. These samples were analyzed for the concentration of 29 PFAS, also after the total oxidizable precursor (TOP) assay. Enrichment factors were defined as the PFAS concentration in the foam divided by the PFAS concentration in the influent. Although foam partitioning did not lead to decreased ∑PFAS concentrations from influent to effluent in any of the plants, certain long-chain PFAS were removed with efficiencies up to 76%. Moreover, ∑PFAS enrichment factors in the foam ranged up to 10^5^, and enrichment factors of individual PFAS ranged even up to 10^6^. Moving bed biofilm reactors (MBBRs) were more effective at enriching PFAS in the foam than activated sludge processes. Altogether, these high enrichment factors demonstrate that foam partitioning in existing wastewater treatment plants is a promising option for integrated removal. Promoting foam formation and removing foam from the water surface with skimming devices may improve the removal efficiencies further. These findings have important implications for PFAS removal and sampling strategies at wastewater treatment plants.

## Introduction

Per- and polyfluoroalkyl substances (PFAS) are environmental pollutants mostly known for their high persistency in the environment.^1^ The wide group of PFAS is characterized by the presence of at least one perfluorinated methyl or methylene carbon atom in their molecular structure,^2^ and can be split into long- and short-chain compounds, depending on how many of such −CF_2_– moieties they contain.^3^ Typically, perfluorosulfonic acids (PFSA) are considered to be short-chain for a chain length below or equal to five (C*_n_*F_2*n*+1_SO_3_H, *n* ≤ 5) and perfluorocarboxylic acids (PFCA) for a chain length below or equal to six (C*_n_*F_2*n*+1_COOH, *n* ≤ 6). Several PFAS have been shown to be bioaccumulative and toxic, although data are scarce for most PFAS.^4,5^ PFAS, particularly perfluoroalkyl acids (PFAA), have become ubiquitous in the environment due to their widespread use and high mobility and persistency, with groundwater concentrations ranging from below quantification limits (typically few ng L^–1^) to mg L^–1^, depending on the proximity of contamination sources.^6,7^ Effluents from wastewater treatment plants treating municipal wastewater, industrial process water, or landfill leachate are considered major discharge routes of PFAS,^8,9^ and removing PFAS from these effluents prior to discharge is an important downstream strategy for preventing continued emissions.

PFAS are mostly not removed with conventional primary and secondary treatment steps for wastewater, although some (typically <50%) adsorption to sludge may occur.^10^ On the other hand, precursor PFAS may degrade to PFAA in biological processes, leading to higher PFAA concentrations in effluent than in influent.^11^ Existing wastewater treatment technologies that are effective for the removal of PFAS from water include nanofiltration,^12^ ion exchange,^13^ and adsorption to activated carbon,^14^ but these technologies are energetically and financially costly, particularly for complex matrices. Extensive pretreatment is often required, and in the case of ion exchange and granular activated carbon, regeneration of the sorbent is necessary after approximately every 5000–50,000 bed volumes treated, to prevent breakthrough of especially short-chain PFAS.^15,16^ In Switzerland, adding treatment with powdered activated carbon to all conventional municipal wastewater treatment plants was estimated to increase the costs of wastewater treatment by approximately 30%.^17,18^

Recently, foam fractionation has been established as a comparatively inexpensive and environmentally friendly treatment technology capable of achieving competitive PFAS removal.^19−23^ Foam fractionation exploits the high surface activity that many PFAS share. It works by introducing air bubbles at the bottom of a water column, to which the surface-active PFAS molecules adsorb. If surfactant concentrations in the water are high enough, a PFAS-enriched foam can subsequently be separated from the liquid phase, resulting in a relatively PFAS-free effluent. The sequestered collapsed foamate is only a small fraction (<1 – 10%)^19,24^ of the initial volume and can undergo destructive treatment. Foam fractionation removes long-chain PFAS better than short-chain PFAS because long-chain PFAS have higher air–water sorption coefficients and are thus more surface-active.^21^ Removal efficiencies for long-chain PFAS typically exceed 95%, whereas, e.g., the short-chain perfluorobutanoic acid (PFBA) is often not removed at all.^24,25^

Many existing wastewater treatment plants use aeration as part of their treatment train. Often, the formation of foam is observed on the water’s surface of such treatment processes, which in the case of biological treatment is typically associated with the presence of filamentous microorganisms.^26^ The stability of this foam depends on the presence of three components: air bubbles, surfactants, and hydrophobic particles.^27,28^ Certain undesirable bacteria strains may exacerbate foam formation by producing biosurfactants and by partitioning into the foam and thereby preventing foam collapse.^27−29^ Generally, excessive foam formation is conceived as problematic, since it complicates process control; the foam may overflow onto surrounding areas, and wind-blown foam or aerosols may lead to spreading of contaminants.^26,29^ Conversely, the aim of this study was to investigate whether foam formation could instead be exploited for the integrated removal of PFAS within existing treatment processes.

The study included various wastewater treatment technologies: activated sludge, moving bed biofilm reactor (MBBR), electrocoagulation, and ozonation. Activated sludge is a well-known biological treatment technology for the removal of organic matter and can be extended to include nitrification, denitrification, and biological phosphorus removal.^30^ MBBR is a type of suspended biofilm reactor, where biofilm grows on plastic carriers that are kept in suspension in the reaction tank. Typically, MBBRs are used for organic matter removal, nitrification, and denitrification. Electrocoagulation is a physicochemical treatment process where metal cations are introduced into the water using sacrificial anodes in an electrochemical cell.^31^ These cations form coagulating complexes that destabilize colloidal particles and adsorb contaminants, which are then easily removed from the water by flotation, settling, or filtration. Finally, ozonation is an oxidation process mostly used as a tertiary treatment for the degradation of organic micropollutants such as pharmaceuticals and biocides.^32^

Specific objectives of this study were to (i) measure PFAS concentrations in foam on the surface of wastewater treatment plants; (ii) use these foam concentrations to assess if PFAS enrichment in the foam leads to measurable PFAS removal, and (iii) evaluate if the enrichment of PFAS in the foam is affected by the treatment process, the general chemistry of the influent water, or the presence of oxidizable precursors.

## Materials and Methods

### Site Selection

Ten full-scale wastewater treatment plants from Sweden, The Netherlands, Belgium, Spain, and Australia where foam formation was observed were included in the project. An overview of these plants is given in Table 1. Plants were selected such that a wide variety of water types, treatment processes, geographical locations, and PFAS concentrations were included. The study focused specifically on processes that did not already have PFAS removal or foam formation as part of their design, i.e., no dissolved air flotation or foam fractionation plants were included. “Wastewater treatment” is used as terminology throughout this paper, but treated water types also included landfill leachate, industrial process water, and contaminated stormwater runoff, in addition to municipal wastewater.

**Table 1:** Overview of Wastewater Treatment Plants, MBBR = Moving Bed Biofilm Reactor

site	treatment process	water type	location	time of sampling (2022)
(A)	MBBR	landfill leachate	Sweden	August
(B)	MBBR	industrial process water	Sweden	August
(C)	MBBR	municipal wastewater	Sweden	December
(D)	activated sludge	landfill leachate	The Netherlands	November
(E)	activated sludge	landfill leachate	The Netherlands	October
(F)	activated sludge	municipal wastewater^a^	Belgium	September
(G)	activated sludge	municipal wastewater	Spain	September
(H)	activated sludge	municipal wastewater^b^	Australia	September
(I)	electrocoagulation	stormwater runoff from landfill^c^	The Netherlands	September
(J)	ozonation	municipal wastewater	Sweden	December

a The influent was sampled before sand filtration instead of right before the activated sludge reactor.

b The influent to this site contained approximately 30% industrial wastewater.

c Specifically, the stormwater runoff came from the bottom ash storage area on the landfill.

### Sample Collection

From each treatment plant, four 250 mL of grab samples from each of the following matrices were collected into clean high-density polyethylene (HDPE) or polypropylene (PP) bottles for PFAS analysis: the influent to the foaming process (i.e., MBBR, activated sludge, electrocoagulation, or ozonation), the effluent from the foaming process, and water from approximately 1 m under the foam. Additionally, samples from the foam were taken for PFAS analysis, which was collapsed to liquid phase prior to transport, and 1 L of influent to the foaming process was sampled for general chemistry analysis. Foam samples were collected into HDPE or PP containers by scooping up foam from the water surface and were either left to collapse spontaneously or forced to collapse under warm air with a heat gun or by mechanical stirring. The sampled volume of collapsed foam differed per plant, and the volume that was analyzed is given in Table SI 3. The reachability of the foam and the availability of equipment were different for each plant, so the exact equipment used for foam sampling varied between plants as well. Samples from sites (C) to (I) were shipped cooled to the Swedish University of Agricultural Sciences (SLU), Uppsala, Sweden, where they were analyzed in our laboratory. Sites (A) and (B) were located sufficiently close to enable manual transport of the samples. All PFAS samples were stored at ∼4 °C until extraction, which was always done within 2 weeks after arrival in the laboratory. General chemistry samples were stored at −20 °C prior to shipment to ALS Scandinavia, Danderyd, Sweden, for analysis of metals, fluoride, chloride, phosphorus, nitrogen, and total organic carbon (TOC) concentrations and conductivity, pH, and turbidity.

### Analysis

Three of the water samples for each matrix type (influent, effluent, and water under the foam) were filtered, extracted, and analyzed for the concentrations of 29 PFAS (for full names, see Table SI 1), using a previously established method.^24^ In brief, 125 mL samples were sonicated, filtered through glass microfiber filters (Whatman, 0.7 μm pore size, China), and spiked with 5 ng absolute each of 20 internal standards,^24^ and solid-phase extraction (SPE) was performed using Oasis WAX cartridges (6 mL, 150 mg, 30 μm, Waters, Ireland). Extracts were analyzed on a SCIEX Triple Quad 3500 ultraperformance liquid chromatography–tandem mass spectrometry (UPLC-MS/MS) system, using scheduled multiple reaction monitoring (sMRM) mode with negative electrospray ionization. An eleven-point calibration curve was used, with concentrations between 0.05 and 900 ng mL^–1^, see SI Section 1.2. Because of this wide concentration range, seven PFAS were quantified based on quadratic calibration curves instead of linear, see SI Table 2. The quantification was done according to the isotope dilution method and using the IS, as described previously.^24^ For compounds with linear as well as branched isomers, only summed concentrations were measured. Median relative standard deviations of the triplicates’ ∑PFAS concentrations were 2.6, 7.7, 3.7, and 3.5% for all influent, effluent, water under the foam, and foam samples, respectively.

To minimize matrix effects and stay in the concentration range of the calibration curve, foamate samples were diluted prior to filtration and extraction. Depending on the aqueous PFAS concentrations at the corresponding treatment plant, a foamate volume between 0.25 and 10 mL was diluted with Milli-Q water to a total volume of 50 mL. The exact volume of foamate that was used for each treatment plant is given in Table SI 3. For site (A), a foamate dilution series was analyzed to confirm that the reported concentrations were independent of the volume used. These results are given in Table SI 4, and quality control results of spiked samples are given in Table SI 5. All foamate concentrations are given per unit volume of collapsed foam, i.e., in the liquid phase.

On the fourth sample for each sample type, i.e., influent, effluent, water under the foam, and foamate, a total oxidizable precursor (TOP) assay was performed using a method developed by Houtz and Sedlak.^33^ The TOP assay is a tool to quantify concentrations of precursors, which can be transformed to PFAA upon oxidation.^33,34^ In brief, 2 g of potassium persulfate (K_2_S_2_O_8_, Sigma-Aldrich) and 1.9 mL of 10 M sodium hydroxide (NaOH, Sigma-Aldrich) were added to each 125 mL sample. For the 50 mL diluted foamate samples, 0.8 g of K_2_S_2_O_8_ and 0.76 mL of 10 M NaOH were used instead. Samples were placed in a water bath at 80 °C for 6 h, cooled in ice, and gradually adjusted to a pH of 6–8 by adding 30% hydrogen chloride (HCl, Merck, Germany). Filtration, SPE, and UPLC-MS/MS analysis were subsequently done, as described above.

Seven laboratory blanks (cartridges preconditioned, IS-spiked, and eluted without the addition of any sample), five Milli-Q blanks (50 mL of Milli-Q extracted and analyzed as normal samples), and seven TOP blanks (125 mL of Milli-Q on which a TOP assay was performed) were included. Method limits of quantification (LOQs) were calculated based on the detected concentrations in the blanks, see SI Section 1.1, and are given in Table SI 1. For all PFAS, LOQs ranged between 0.4 and 4 ng L^–1^ for the water and between 5 and 2000 ng L^–1^ in the foamate.

### Data Handling

All data handling, plotting, and statistical analyses were done in Matlab, version 2020b. PFAS removal efficiencies (RE) were calculated as per eq 1, with *C*_In_ and *C*_Ef_ being the mean concentration of the influent and effluent samples, respectively (*n* = 3 for each). PFAS enrichment factors (EF) were calculated using eq 2, with *C*_foam_ being the mean concentration foamate samples (*n* = 3).
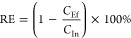
1
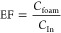
2

PFAS concentrations and enrichment factors were log-transformed prior to any correlation analyses. When a PFAS was not detected above the LOQ in any of the samples at a site, its concentration was set to zero for all samples from that site. When a PFAS was detected in at least one sample from a site, concentrations below the LOQ were set to half the LOQ unless stated otherwise. To illustrate the range of uncertainty caused by the inclusion of non-detect concentrations, certain figures have been repeated in the SI, with non-detect concentrations set to zero or the LOQ instead (Figures SI 1 and 2 and 4–7). For the correlation analysis on the metal, chloride, and fluoride concentrations, concentrations below the LOQ were set to half the LOQ. Any correlations with mercury were ignored because of the high proportion (50%) of non-detects.

## Results and Discussion

### ∑PFAS Removal Due to Foam Partitioning

**Figure 1:**
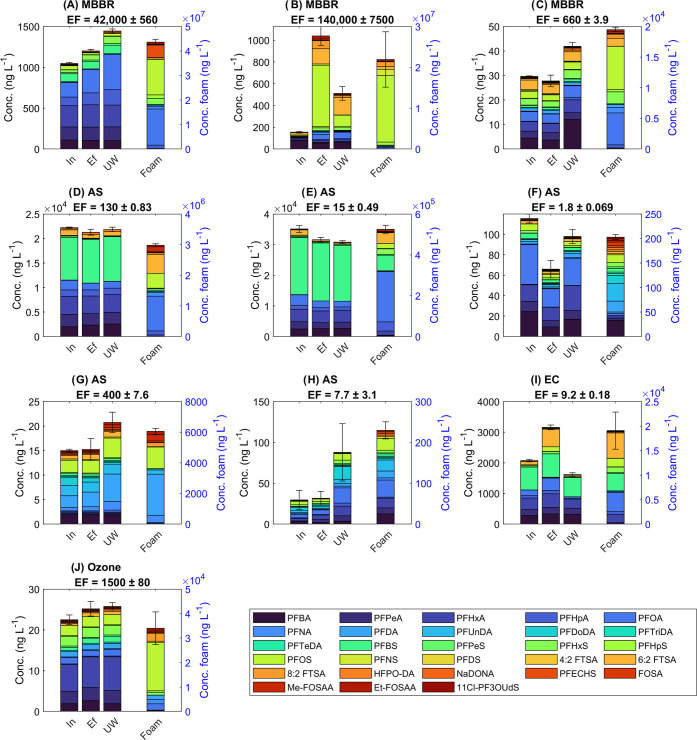
PFAS concentrations (ng L^–1^) in the influent (In), effluent (Ef), water under the foam (UW), and foamate (Foam) for all treatment plants included in the study (see Table 1, labels of the subplots correspond to the site identifiers). MBBR = moving bed biofilm reactor, AS = activated sludge, EC = electrocoagulation, and Ozone = ozonation. Foamate concentrations are on a different scale, represented by the blue *y*-axis on the right. Titles give the enrichment factor (EF), calculated as per eq 2, with the corresponding standard deviation. Error bars represent the standard deviation (*n* = 3) over the ∑PFAS concentrations for each sample type. Repeated figures with concentrations below LOQ set to zero or the LOQ, instead of 0.5·LOQ, are given in Figures SI 1 and 2. For the mean concentration data, see Table SI 6.

Out of the 29 targeted PFAS, all except 9Cl-PF3ONS were detected in at least one sample. As visualized in Figure 1, the concentrations and compositions of the 28 detected PFAS varied widely between the different treatment plants, but PFAS concentrations were consistently higher in the foamate than in the influent to the treatment process. The only treatment plant with considerable removal of ∑PFAS from influent to effluent was site F, the activated sludge municipal WWTP in Belgium, with a mean ∑PFAS removal of 43%. However, this was coincidentally also the plant with the lowest ∑PFAS enrichment in the foam, and the PFAS removal from influent to the water under the foam was only 15%. Therefore, the PFAS removal here was probably not caused by accumulation of PFAS in the foam but by PFAS adsorption to sludge.

In all other plants, the ∑PFAS concentrations from influent to effluent were either unchanged (<10% difference, sites C, D, E, G, and H) or increased (sites A, B, I, and J). For site J, the 13% higher PFAS concentrations in the effluent may be due to precursor degradation, since increased PFAS concentrations were measured in the influent after the TOP assay (see Figure SI 3), and the ozonation treatment used at this plant may result in precursor degradation.^35^ Additionally, for all sites, variability in the treated water may have resulted in higher effluent than influent concentrations, since concentrations were based on grab samples during one occasion rather than time-integrated samples. The effluent water at site B was still rather foamy, so it is possible that the effluent samples included some foam, leading to the 570% higher effluent concentrations compared to the influent. This explanation is especially probable considering the extremely high mean ∑PFAS concentrations in the foamate at site B of 22 mg L^–1^.

Electrocoagulation has been found to remove PFAS on a laboratory scale, which is generally believed to occur because PFAS adsorb to the formed metal flocs.^36,37^ It has even been shown that PFAS partitioning into the foam formed during electrocoagulation plays a role in the removal mechanism, particularly when the electrocoagulation process is operated at a high current density.^38^ In the current study, no ∑PFAS removal from influent to effluent was found in plant I, a full-scale electrocoagulation reactor with iron electrodes (Figure 1I). PFAS removal by sorption to the iron flocs was not found either, since the effluent concentrations were higher than the influent concentrations.

Overall, while there is clear evidence of PFAS enrichment in the foam, this mechanism did not seem to result in considerable ∑PFAS removal from the influent in any of the investigated treatment plants. A reason for this could be that the foam was not actually removed at any of the plants, but instead left on the water surface to eventually collapse back into the effluent. To test this hypothesis, water under the foam was also sampled at all plants, and >10% ∑PFAS removal from the influent to the water under the foam was found at sites E (12%) and I (22%), as well as the aforementioned site F (15%). While these removal efficiencies are still not very high, they give some indication that removing the foam from the water surface may increase the ∑PFAS removal efficiency, leading to lower concentrations in the water under the foam as well as the effluent.

An additional explanation for the lack of ∑PFAS removal from influent to effluent, despite the high enrichment factors, is the possible discrepancy in retention time between foam and water. All treatment plants included in the study were in continuous operation, but none were regularly skimming foam from the surface. Accordingly, particularly in plants where a high buildup of foam was observed, the high PFAS concentrations in the foam probably reflected the PFAS removal over multiple hydraulic retention times (HRT). Because the foam volumes or flow rates were not measured, it is impossible to quantify the importance of this source of uncertainty. Nonetheless, for plant B, which had the highest enrichment factor, the dosing of anti-foaming agent was stopped less than one HRT before sampling the foam, and foam buildup occurred very quickly. Thus, while long-term accretion of PFAS in the foam layer may have somewhat distorted the measured enrichment factors, it was unlikely to cause the high enrichment measured at all plants.

#### Long-Chain PFAA Removal Due to Foam Partitioning

When focusing on the total concentration of long-chain PFAA only, instead of on ∑PFAS concentrations, some removal from influent to effluent was measured at sites C (7%), D (37%), E (46%), and H (12%), in addition to the aforementioned site F (46%). Specifically, at site D, mean perfluorooctane sulfonic acid (PFOS) and perfluorooctanoic acid (PFOA) removal efficiencies were 58 and 30%, respectively. At site E, these were 76% and 36%. For these two sites (D and E), similar long-chain PFAA removal efficiencies were obtained from the influent to the water under the foam, indicating that foam partitioning contributes to the removal mechanism. For site I, an even higher mean ∑long-chain PFAA removal from influent to the water under the foam of 74% was measured, with PFOS and PFOA removal at 57 and 76%, respectively. All of these sites treated water with comparatively high PFAS concentrations, which may have contributed to the measurable concentration decrease of long-chain PFAA.

Attributing the decreased effluent concentrations of long-chain PFAA entirely to accumulation in foam is not realistic. Sites D and E were both activated sludge plants, and since long-chain PFAA are known to be susceptible to adsorption to sludge, this is another probable mechanism for the removal of these compounds. However, since sludge concentrations and sludge production rates were not measured in this study, it was impossible to quantify the relative contribution of PFAS adsorption to sludge. The extent of removal with sludge depends on a combination of operational and solution parameters, e.g., solid retention time, pH, and wastewater composition. In the literature, reported PFAS removal due to adsorption to sludge ranges from zero to nearly full removal, although values > 80% were only achieved in processes with extremely high sludge concentrations and retention times.^10^ Estimating the contribution of sludge adsorption to the total removal is further complicated by the degradation of precursors. In fact, increased PFAA concentrations in the effluent are more commonly reported than decreased concentrations, and analytical techniques such as the TOP assay have only recently become common.^10,34^

#### Implementing Foam Stimulation to Increase Removal

While no evidence of ∑PFAS removal due to foam partitioning was found at any of the plants, there were indications that PFAS removal may occur when foam formation would be stimulated rather than prevented. Lower ∑PFAS concentrations were measured in the water under the foam for some plants, long-chain PFAA were occasionally removed to some extent, and the ∑PFAS concentrations in the foamate were up to 10^5^ times higher than those in the influent. Particularly, these extremely high enrichment factors from influent to foam indicate promise for using foam formation as an integrated removal mechanism. Although PFAS concentrations in the foamate were very high, the total volume of foam compared to the volume of influent water was probably negligible from a mass balance perspective. The reported concentrations were measured in the collapsed, liquid foamate. Actual foam has a very low density, and the foamate thus constitutes only a very small fraction of the treated influent volume.

Foam control strategies are common at wastewater treatment plants, particularly chemical methods, such as dosing of anti-foaming agents or disinfectants.^26,39^ Additionally, design decisions may be made to minimize foam formation, e.g., lowering the sludge retention time in activated sludge, operating at lower aeration rates, or implementing anoxic, anaerobic, or aerobic selector systems.^26^ Such selector systems aim to provide unfavorable conditions for the growth of foam-causing filamentous microorganisms.^39^ If tanks are mechanically stirred, this may also lead to foam collapse instead of buildup. When foam formation is to be exploited as a PFAS removal technology, foam removal devices that skim foam from the water surface should be installed instead. Designing the treatment process to stimulate foam formation rather than prevent it could possibly enhance the removal efficiency significantly as compared to the values reported here.

An attempt to quantify the increase in foam formation that would be required for the significant removal of long-chain PFAA is presented in SI Section 2.4. The derivation of the equation used for this quantification, eq SI 1, relied on several assumptions.^40^ Most importantly, adsorption to sludge and reactive transformation of PFAA were ignored, and the foam wetness was assumed to remain constant with increasing foam fraction, i.e., the ratio of foamate flow over influent flow. In other words, the increase in foamate flow was assumed to be entirely due to an increase in foam film surface area available for PFAS sorption, rather than an increase in liquid fraction of the foam. The need for these assumptions inherently compromises the reliability of the analysis, and the results should be seen as rough estimations only, but they indicated that high removal of long-chain PFAA with foam might be possible at certain plants. Specifically, a sevenfold increase in foam fraction could result in a ∑long-chain PFAA removal of ∼80% at sites D and E. Additionally, at site J, a removal of >99% would possibly require a foam fraction of only 3%. More detailed results are given in Figure SI 6.

#### Enrichment Factors for Individual PFAS

**Figure 2:**
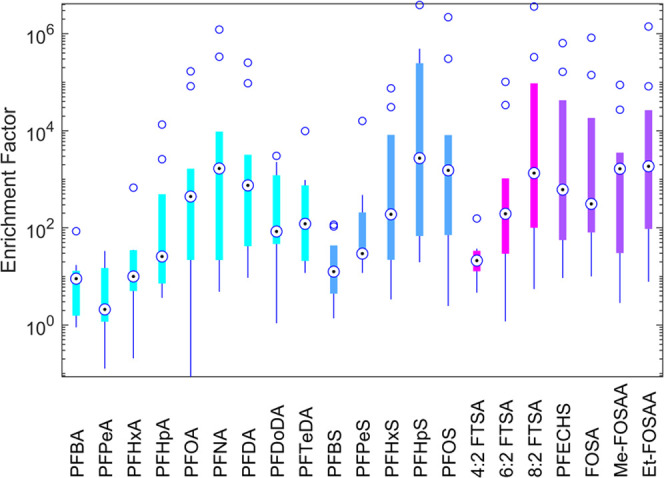
Box plot of enrichment factors across the different sites for individual PFAS. PFCA are colored light blue, PFSA dark blue, FTSA magenta, and the other PFAS purple. Compounds of the same class have an increasing molecular weight from left to right. Only PFAS that were detected at at least eight of the sites were included, i.e., 8 ≤ *n* ≤ 10. The bottom and top of each box represent the 25th to the 75th percentile, respectively. The black dot encircled in blue represents the median, and whiskers go to the most extreme data points, excluding outliers. Outliers (blue circles) are values more than 1.5 interquartile range from the bottom or top of the box. Full names of all PFAS are given in Table SI 1.

From the literature on foam fractionation, it is known that long-chain PFAS are more susceptible to foam partitioning than short-chain PFAS.^24,25,41^ As visualized in Figure 2, the enrichment factors found across all treatment plants included in this study were indeed generally higher for longer-chained compounds. Nonetheless, the spread in enrichment factor per PFAS was very wide. For example, the enrichment factor of perfluoroheptane sulfonic acid (PFHpS) ranged over six orders of magnitude. The study included a variety of water matrices, treatment processes, and plant designs, and PFAS, dissolved solids, and organic carbon concentrations also varied widely between the ten sites. The density of the foam was not measured but was observed to vary between the plants. Because of all of these changing variables, it is unsurprising that a high variability in the enrichment factors was found.

#### Effect of Treatment Process and Water Type

Over the ten treatment plants, ∑PFAS concentrations in the influent, effluent, and water under the foam correlated strongly with each other (all *r* > 0.97, all *p* < 10^–5^). Conversely, foamate ∑PFAS concentrations only correlated significantly with effluent concentrations (*r* = 0.63, *p* = 0.049) but not with ∑PFAS concentrations in the influent or the water under the foam. Enrichment factors correlated significantly with foamate ∑PFAS concentrations (*r* = 0.76, *p* = 0.01) but not with ∑PFAS concentrations in any of the other sample types. Moreover, enrichment factors did not correlate significantly with the fraction of short-chain or long-chain PFAA of the influent ∑PFAS concentrations (all *p* > 0.05). These correlations indicate that the magnitude of PFAS enrichment in foam is relatively independent of the aqueous PFAS concentrations and composition profiles, but instead depends on foam characteristics. The correlations described here were similar for all inclusion methods of non-detect concentrations.

**Figure 3:**
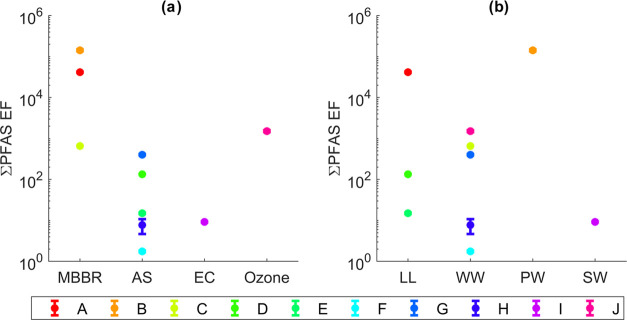
∑PFAS enrichment factors (EF) grouped by (a) treatment process and (b) water type. MBBR = moving bed biofilm reactor, AS = activated sludge, EC = electrocoagulation, Ozone = ozonation, LL = landfill leachate, WW = wastewater, PW = process water, and SW = stormwater runoff from landfill bottom ash collection site. The letters in the legend correspond to the site identifiers given in Table 1. Error bars represent the standard deviation (sd) within the EF for each plant (*n* = 3 for foamate as well as influent concentrations) but are difficult to see for all plants except H because the sd was relatively small. Repeated figures with concentrations below the LOQ set to zero or the LOQ, instead of 0.5·LOQ, are given in Figures SI 7 and 8.

Figure 3a shows the ∑PFAS enrichment factor of all sites grouped by treatment process. MBBRs appear particularly effective at enriching PFAS in foam, with significantly higher enrichment factors than activated sludge processes (1-way ANOVA, *p* = 0.04, followed by Tukey’s honestly significant difference procedure). This difference was not significant (*p* > 0.05) when all non-detect concentrations were set to zero (see also Figure SI 6). Possibly, this higher enrichment is because MBBRs do not contain suspended sludge, contrary to activated sludge processes. The presence of suspended sludge may have decreased the PFAS enrichment by adsorbing the PFAS prior to its incorporation in the foam. Similarly, the foam from activated sludge plants generally contained a lot of floating sludge. This floating sludge layer may have prevented the foam from building up, thereby preventing the formation of dry, highly PFAS-enriched foam.

The ozonation plant (J) also had a very high mean enrichment factor of 1500. In a study by Dai et al., higher PFAS removal efficiencies were found with ozonated air fractionation than with conventional foam fractionation.^42^ Dai et al. hypothesized that an increased PFAS affinity for a gas bubble surrounded by hydroxyl radicals formed in the ozonation process boosted the accumulation of PFAS in the ozonated foam, due to the affinity of the hydrophilic PFAS head groups to these radicals. Possibly, this effect also played a role in the high enrichment measured in the foam from site J. Additionally, precursor degradation may have increased the foam concentrations even further.^34^ The influent, effluent, and water under the foam concentrations all increased after the TOP assay, but the foamate concentration did not (Figure SI 3). Since the foam is formed by being in contact with ozone bubbles, the vast majority of the precursors had probably already degraded in the foam, thereby increasing the measured target PFAS concentrations. In contrast, the influent still contained precursors, which were not measured in the target PFAS analysis. When considering the concentrations after the TOP assay, the enrichment factor was thus only 620 instead of 1500. A similar effect may have played a role at sites C, G, and H, which also had lower enrichment factors after the TOP assay, but here, precursors would have been oxidized by oxygen or biodegradation rather than ozone.

#### Effect of General Chemistry

There were no clear differences in enrichment factor between the different water types, as illustrated in Figure 3b. Moreover, no significant correlations between the enrichment factor and any of the tested general chemistry parameters were found (Pearson’s r, all *p* > 0.05). Removal efficiencies of ∑PFAS (*r* = 0.67, *p* = 0.049) and ∑long-chain PFAS (*r* = 0.77, *p* = 0.02) only correlated significantly with total phosphorus concentrations but not with any of the other general chemistry parameters. The removal efficiencies from site B were excluded from the correlation analyses with general chemistry, since these were strongly negative (see Figure 1B) and thus not realistic. Altogether, this lack of strong correlations indicates that PFAS enrichment and removal were mostly independent of the measured general chemistry parameters in the influent. This differs from foam fractionation results reported in the literature, where higher concentrations of certain metal ions and higher conductivity were found to correlate with a higher PFAS removal.^21,22^ A full overview of the general chemistry results is given in Table SI 7.

#### Role of Precursors

Precursors degrade to PFCA in the TOP assay,^33^ so the fraction of PFCA is expected to increase after the TOP assay. The fraction of PFCA in the total PFAS concentrations was indeed higher after the TOP assay for 90% of the samples (total *n* = 40). The only samples with a decreased fraction of PFCA were the effluents of site B (−19%), E (−7%), and H (−4%) and the water under the foam of site F (−2%). At site B, PFOS concentrations in the effluent nearly doubled after the TOP assay (see Figures 1 and SI 3), which caused a decreased fraction of PFCA after the TOP assay. Probably, PFOS precursors were present at this site in high concentrations. For the remaining sites, the relative decrease in PFCA concentrations was probably due to measurement uncertainties combined with generally low precursor concentrations in these water types.

As mentioned previously, lower enrichment factors were measured after the TOP assay at sites C, G, H, and J, probably because of precursor degradation in the foam at the treatment site. In contrast, higher enrichment factors were measured after the TOP assay at sites B (22% increase), D (39%), E (44%), F (260%), and I (53%) (see Figures 1 and SI 3). When more precursors are present in the foam than in the influent, precursors are enriched in the foam, and a higher enrichment factor will be measured after the TOP assay. Accordingly, these higher enrichment factors indicate that precursors were enriched in the foam at sites B, D, E, F, and I. Since some well-known precursors are used as surfactants (e.g., perfluoroalkyl phosphonic acids (PFPAs) and polyfluoroalkyl phosphoric acid esters (PAPs)),^3,33^ it is unsurprising that oxidizable precursors were susceptible to enrichment in the foam.

## Environmental Implications

This study aimed to evaluate the potential of using existing wastewater treatment plants as foam fractionators for the removal of PFAS. The results were twofold. On the one hand, no removal of ∑PFAS was measured at any of the investigated sites that could be attributed to foam partitioning. On the other hand, the high enrichment factors of PFAS in foam show promise for combining conventional wastewater treatment with foam fractionation, and >35% removal of long-chain PFAA was measured at two of the investigated sites. A preliminary mass balance analysis showed that >80% long-chain PFAA removal with foam may be achievable at reasonable (<5%) foam fractions in certain plants. Full-scale attempts to implement foam skimming are required to assess the viability of integrated foam fractionation for PFAS removal and to see if higher removal efficiencies can be achieved.

Despite its exploratory nature, the study showed that MBBRs may be particularly effective for the separation of PFAS in foam, but artificially increasing the foam formation would be necessary to achieve quantifiable PFAS removal. Combining stimulated foam formation with full-scale foam removal in an MBBR would thus be a fruitful area for future work. Additionally, it would be interesting to investigate if this high PFAS enrichment in foam is also found in other compact biofilm-based processes, such as aerobic granular sludge (Nereda)^43^ and membrane bioreactors (MBRs).^44,45^ Finally, further research should include measurement of foam characteristics to better understand the role these variables play in the enrichment of PFAS. Various methods have been developed to quantifiably evaluate foam on wastewater, such as measuring foam rating, volume, foam power, foam stability, and scum index,^46^ and these may generate further insight into what determines the level of PFAS enrichment in foam.

A downside of using existing plants for the removal of PFAS, rather than specifically designed systems, is that the plant performance in terms of chemical oxygen demand (COD), nutrient, or micropollutants removal must not be compromised. Each process investigated in this study was designed for a specific treatment objective, and it should be ensured that artificially promoting foam formation for the removal of PFAS does not influence the achievement of that objective. This may limit process options such as modifying the aeration rate or dosing surfactants that are available in traditional foam fractionation for the removal of PFAS.

In addition to the implications related to integrated PFAS removal, the findings presented here also have important implications for sampling strategies. When influent and effluent to a wastewater treatment process are sampled to determine PFAS concentrations, the inclusion or exclusion of foam from the sample may affect the measured concentrations significantly. This is particularly important when grab samples are taken from the water surface, as this increases the likelihood of including foam in the sample, thus overestimating the PFAS concentrations. There is, therefore, a definite need for standardized sampling methods when effluent concentrations are measured for checking the compliance with permitted concentrations.

## Supporting Information Available

Overview of LOQs; volumes used for the extraction of foamate and quality control results; Figures 1 and 3 repeated with alternative handling of concentrations below the LOQ; mean PFAS concentrations; TOP assay results; approximate quantification of the required foam fraction for increased PFAS removal and general chemistry results (PDF)
