# Supplementary material for: Integrated Treatment of Per- and Polyfluoroalkyl Substances in Existing Wastewater Treatment Plants—Scoping the Potential of Foam Partitioning
Source: ACS ES T Eng. 2023 Aug 1;3(9):1276–85. doi: 10.1021/acsestengg.3c00091 (PMC10496112; doi:10.1021/acsestengg.3c00091)
Supplement: Supplementary file 1 — ee3c00091_si_001.pdf [file ee3c00091_si_001.pdf]

Supplementary information to:
Integrated treatment of per- and polyfluoroalkyl substances in existing water treatment plants – scoping the potential of foam partitioning

Sanne J. Smith[1*], Chantal Keane[2], Lutz Ahrens[1] and Karin Wiberg[1]

[1]Department of Aquatic Sciences and Assessment, Swedish University of Agricultural Sciences (SLU), P.O. Box 7050, SE-750 07, Uppsala, Sweden
[2]University of Queensland, Queensland alliance for Environmental Health Sciences, Woolloongabba, QLD 4102, Australia
E-mail contact: sanne.smith@slu.se

1. Supplementary methods

1.1 Limits of quantification

Method limits of quantification (LOQs) for the sample extracts were calculated using Equation SI 1, with $\langle C_{blank} \rangle$ and $sd_{C_{blank}}$ the mean and standard deviation of the extract concentrations in the blanks (*n = 12*), respectively. Outlying blank concentrations (defined as being more than three standard deviations away from the mean) were removed prior to LOQ calculations, and concentrations below the instrument quantification limit (0.05 ng mL^{-1}) were set to 0.05 ng mL^{-1}. For the calculation of the LOQ of TOP assay samples, the concentrations in the TOP blanks (*n* = 7) were used instead. Because the volume of foamate extracted was lower than that of water, LOQs for water samples were lower than those for foam samples. The LOQs as given in Table SI 2 were converted based on the volume of sample extracted to give the LOQ in each sample.

$$LOQ = \langle C_{blank} \rangle + 10 \cdot sd_{C_{blank}}$$

Equation SI 1

Table SI 1: LOQs in sample extracts and extracts of samples after the TOP assay. The LOQs in the samples varied based on the extracted sample volume. An extract LOQ of 0.05 ng mL^{-1} corresponds to a sample LOQ of 0.4 ng L^{-1} for a sample of 125 mL, 5 ng L^{-1} for a sample of 10 mL (the highest foamate volume extracted) and 200 ng L^{-1} for a sample of 0.25 mL (the lowest foamate volume extracted).

Compound	LOQ normal extracts (ng mL^{-1})	LOQ TOP extracts (ng mL^{-1})
**Perfluorobutanoic acid (PFBA)**	0.50	1.5
**Perfluoropentanoic acid (PFPeA)**	0.05	0.85
**Perfluorobutane sulfonate (PFBS)**	0.12	0.06
**Perfluorohexanoic acid (PFHxA)**	0.05	0.71
**4:2 Fluorotelomer sulfonic acid (4:2 FTSA)**	0.05	0.05
**hexafluoropropylene oxide dimer acid (HFPO-DA)**	0.05	0.05
**Perfluoropentane sulfonate (PFPeS)**	0.05	0.05
**Perfluoroheptanoic acid (PFHpA)**	0.05	0.05
**4,8-dioxa-3H-perfluorononanoic acid (NaDONA)**	0.05	0.05
**Perfluorohexane sulfonate (PFHxS)**	0.13	0.21
**Perfluorooctanoic acid (PFOA)**	0.05	1.1
**6:2 Fluorotelomer sulfonate (6:2 FTSA)**	0.05	1.1
**Perfluoroheptane sulfonate (PFHpS)**	0.05	0.05
**Perfluoroethyl-cyclohexane sulfonate (PFECHS)**	0.05	0.05
**Perfluorononanoic acid (PFNA)**	0.05	0.05
**Perfluorooctane sulfonamide (FOSA)**	0.05	0.05
**Perfluorooctane sulfonate (PFOS)**	0.24	0.35
**Perfluorodecanoic acid (PFDA)**	0.34	0.37
**8:2 Fluorotelomer sulfonate (8:2 FTSA)**	0.05	0.05
**9-chloro-hexadecafluoro-3-oxanonane sulfonate (9Cl-PF3ONS)**	0.07	0.08
**Perfluorononane sulfonate (PFNS)**	0.05	0.05
**Perfluoroundecanoic acid (PFUnDA)**	0.54	0.24
**N-methyl-perfluorooctane sulfonamido acetic acid (MeFOSAA)**	0.05	0.05
**N-ethyl-perfluorooctane sulfonamido acetic acid (EtFOSAA)**	0.05	0.05
**Perfluorodecane sulfonate (PFDS)**	0.05	0.05
**Perfluorododecanoic acid (PFDoDA)**	0.09	0.11
**11-chloro-eicosafluoro-3-oxaundecane-1-sulfonic acid (11Cl-PF3OUdS)**	0.05	0.05
**Perfluorotridecanoic acid (PFTriDA)**	0.05	0.05
**Perfluorotetradecanoic acid (PFTeDA)**	0.05	0.05

1.2 Calibration curve

The calibration curve concentrations were 0.05, 0.1, 0.5, 1, 5, 10, 50, 100, 250, 500 and 900 ng mL^{-1}. A calibration curve was run before and after all samples from one site. Because the variation in PFAS concentrations between the different samples and the different sites was large, it was necessary to use a calibration curve that included a wide range of concentrations. For some compounds, this curve was not linear over the full concentration range. When necessary, certain points of the calibration curve were excluded or the regression was changed to quadratic, to ensure a good fit of all calibration curve points ($R^2 \geq 0.99$). All compounds with excluded concentration points or a non-linear regression are shown in Table SI 2. Extract concentrations outside of the range of the calibration curve (> 900 ng mL^{-1}) were extrapolated based on the curve's regression equation. This only applied to at most three compounds at four sites, with the concentrations always being acceptably close to the highest included standard concentration.

Table SI 2: Compounds with changed calibration curve regression methods. For compounds not included in this table, all concentration points were included and a linear regression was used. ◊PFBA: the 900 ng mL^{-1} point was included for site D, because PFBA concentrations were >500 ng mL^{-1} in some of the sample extracts of this site. ○4:2 FTSA and NaDONA: R^2 was 0.98 for the calibration curve regression of one site (I and A, respectively) for each of these compounds.

Component	Regression	Excluded concentrations (ng mL^{-1})
**PFBA◊**	Quadratic	900
**PFPeA**	Quadratic	
**4:2 FTSA○**	Quadratic	250, 500, 900
**HFPO-DA**	Quadratic	500, 900
**NaDONA○**	Linear	250, 500, 900
**6:2 FTSA**	Linear	900
**FOSA**	Linear	500, 900
**8:2 FTSA**	Quadratic	900
**9Cl-PF3ONS**	Quadratic	500, 900
**PFECHS**	Quadratic	
**Me-FOSAA**	Linear	900
**PFDoDA**	Linear	900
**11Cl-PF3OUdS**	Linear	250, 500, 900
**PFTriDA**	Linear	250, 500, 900
**PFTeDA**	Linear	250, 500, 900

1.3 Foamate extraction

Table SI 3: Volumes of foamate extracted for each site. The foamate was always diluted to 50 mL with Milli-Q water prior to extraction.

Site	Volume of foamate (mL)
**A**	0.25
**B**	0.25
**C**	5.00
**D**	0.25
**E**	0.25
**F**	7.50
**G**	7.50
**H**	10.0
**I**	1.00
**J**	2.50

Table SI 4: Mean PFAS concentrations (µg L⁻¹) in foamate from site A for different extracted volumes (n = 2 for each volume), always diluted to a total volume of 50 mL. In the calculation of the relative standard deviation (%), samples for which both concentrations were below the LOQ were excluded. When the concentration in one duplicate was above the LOQ, the concentration in the duplicate below the LOQ was set to the LOQ. For some compounds, concentrations could not be determined at high extracted volumes, because matrix interference pushed the compound peak out of the sMRM window. Cells have been left blank when this was the case. Relative errors were always low, except when the concentrations were very close to the quantification limits (e.g. 4:2 FTSA, PFDoDA and PFTeDA) and thus had a high analytical uncertainty.

Compound	Extracted Volume					Relative standard deviation (%)
	0.1 mL	0.25 mL	0.5 mL	1 mL	2.5 mL	
	Concentration ($\mu g\ L^{-1}$)					
**PFBA**	<5.0	<2.0	<1.0	0.69	0.62	9%
**PFPeA**	1.5	1.4	1.3	1.5	1.5	5%
**PFBS**	13	11	11	11	12	7%
**PFHxA**	180	170	180	190	190	4%
**4:2 FTSA**	0.61	0.22	0.16	0.12	0.11	83%
**HFPO-DA**	1.3	1.1	1.1	1.3	1.1	14%
**PFPeS**	190	260	300	360	400	26%
**PFHpA**	1300	1300	1400	1200	1200	8%
**NaDONA**	0.83	0.61	0.60	0.58	0.48	28%
**PFHxS**	3000	3100	2700	2700	2100	13%
**PFOA**	16000	14000	12000	9800		20%
**6:2 FTSA**	550	620	530	510		11%
**PFHpS**	1400	1500	1500			4%
**PFECHS**	3500	3500	3400			3%
**PFNA**	1100	1100	1000	1000		6%
**FOSA**	200	190	170	170		9%
**PFOS**	19000	17000	16000			8%
**PFDA**	480	520	490	470		8%
**8:2 FTSA**	79	78	63			11%
**9Cl-PF3ONS**	<0.74	<0.30	<0.15	0.08		N/A
**PFNS**	69	79				13%
**PFUnDA**	16	19	13	12		22%
**Me-FOSAA**	150	150				3%
**Et-FOSAA**	730	620				11%
**PFDS**	3.9	3.3	3.3			13%
**PFDoDA**	7.1	3.9	4.1	2.1		60%
**11Cl-PF3OUdS**	<0.50	<0.20	<0.10	<0.05	<0.02	N/A
**PFTriDA**	0.53	0.26	0.35	0.15	0.07	66%
**PFTeDA**	9.1	0.42	5.4	0.06	0.23	186%

Table SI 5: Recoveries (%, as mean (min – max)) of Milli-Q spiked with 2.5 and 10 ng of each PFAS, and foamate samples spiked with 25 ng of each PFAS. Recoveries well above or below 100 % were often due to high concentrations in the unspiked foamate samples. E.g., when the concentration of an unspiked foamate extract was 250 ng mL^{-1}, a method variability of 10 % may have already caused a recovery of 0 % or 200 %. Recovery of 9Cl-PF3ONS in the foamate samples was consistently low, which indicates that matrix suppression decreased the signal. Since this compound was not detected in any of the samples, it was left out of the data analysis and its low recovery thus did not affect the presented results. The stock solution used to spike the 10 ng Milli-Q samples and the foam samples from Site A was probably contaminated with PFBA, leading to recoveries that were a factor two too high. A different stock solution was used for the remaining samples, in which the recovery of PFBA was always within an acceptable range.

Compound	Spiked Milli-Q (50 mL)		Spiked Foamate (with 25 ng, n = 3 for all)			
	**2.5 ng** (n = 2)	**10 ng** (n = 3)	**Site A**	**Site C**	**Site D**	**Site J**
**PFBA**	117 (113 - 122)	209 (202 - 215)	226 (213 - 245)	97 (96 - 98)	126 (124 - 129)	101 (97 - 109)
**PFPeA**	110 (107 - 112)	142 (138 - 145)	145 (139 - 152)	99 (97 - 101)	118 (116 - 120)	106 (104 - 107)
**PFBS**	109 (106 - 113)	118 (117 - 119)	126 (122 - 133)	100 (98 - 103)	120 (116 - 124)	112 (108 - 114)
**PFHxA**	111 (107 - 114)	111 (106 - 119)	149 (137 - 164)	127 (124 - 130)	124 (118 - 128)	125 (122 - 128)
**4:2 FTSA**	102 (91 - 113)	106 (98 - 115)	116 (108 - 127)	80 (78 - 82)	100 (99 - 101)	88 (81 - 95)
**HFPO-DA**	102 (98 - 105)	114 (97 - 130)	237 (222 - 255)	189 (176 - 207)	175 (156 - 188)	170 (160 - 183)
**PFPeS**	94 (91 - 98)	103 (98 - 105)	248 (229 - 275)	88 (84 - 92)	117 (111 - 121)	78 (73 - 81)
**PFHpA**	100 (94 - 105)	108 (93 - 116)	143 (-3 - 299)	111 (108 - 114)	125 (103 - 146)	136 (130 - 144)
**NaDONA**	136 (134 - 138)	138 (123 - 152)	146 (141 - 154)	169 (149 - 195)	174 (137 - 195)	210 (195 - 230)
**PFHxS**	81 (81 - 81)	88 (87 - 89)	130 (-40 - 433)	73 (69 - 77)	92 (87 - 98)	78 (75 - 81)
**PFOA**	107 (95 - 118)	112 (102 - 124)	658 (19 - 1064)	111 (98 - 121)	217 (152 - 272)	124 (122 - 128)
**6:2 FTSA**	114 (107 - 121)	116 (110 - 126)	110 (57 - 164)	100 (97 - 103)	184 (174 - 192)	113 (110 - 114)
**PFHpS**	123 (120 - 125)	130 (124 - 137)	260 (111 - 361)	134 (132 - 136)	132 (127 - 136)	129 (114 - 143)
**PFECHS**	75 (73 - 77)	100 (93 - 109)	-3222 (-5431 - 943)	130 (127 - 133)	144 (140 - 147)	122 (115 - 130)
**PFNA**	88 (83 - 93)	112 (105 - 120)	262 (157 - 377)	108 (102 - 111)	123 (120 - 128)	107 (100 - 118)
**FOSA**	102 (100 - 104)	113 (100 - 128)	124 (96 - 151)	102 (99 - 104)	118 (115 - 121)	100 (94 - 108)
**PFOS**	74 (74 - 75)	86 (84 - 89)	439 (-1282 - 1572)	68 (41 - 104)	153 (122 - 195)	62 (-32 - 173)
**PFDA**	73 (72 - 74)	80 (78 - 82)	236 (166 - 300)	103 (96 - 109)	105 (96 - 120)	91 (83 - 106)
**8:2 FTSA**	113 (111 - 114)	130 (126 - 134)	141 (125 - 152)	77 (74 - 81)	99 (93 - 106)	89 (77 - 105)
**9Cl-PF3ONS**	91 (87 - 94)	122 (119 - 126)	49 (48 - 52)	29 (28 - 30)	44 (44 - 45)	30 (29 - 32)
**PFNS**	111 (98 - 124)	127 (125 - 130)	378 (334 - 405)	120 (119 - 120)	151 (145 - 156)	117 (111 - 125)
**PFUnDA**	51 (49 - 53)	60 (56 - 64)	154 (150 - 162)	107 (102 - 111)	100 (90 - 105)	97 (93 - 105)
**Me-FOSAA**	111 (110 - 112)	118 (102 - 130)	129 (97 - 150)	104 (99 - 111)	133 (109 - 152)	98 (92 - 109)
**Et-FOSAA**	102 (100 - 104)	114 (113 - 117)	149 (60 - 282)	91 (89 - 93)	118 (112 - 124)	91 (84 - 101)
**PFDS**	97 (95 - 99)	109 (105 - 114)	434 (401 - 459)	89 (86 - 92)	151 (147 - 154)	97 (95 - 101)
**PFDoDA**	110 (104 - 115)	126 (122 - 132)	202 (189 - 214)	134 (131 - 137)	131 (123 - 137)	130 (118 - 146)
**11Cl-PF3OUdS**	69 (63 - 76)	92 (87 - 95)	285 (274 - 302)	105 (104 - 107)	77 (74 - 79)	73 (66 - 79)
**PFTriDA**	112 (99 - 124)	145 (141 - 151)	231 (190 - 288)	88 (87 - 89)	131 (114 - 149)	148 (121 - 198)
**PFTeDA**	91 (82 - 101)	111 (103 - 116)	137 (108 - 175)	91 (85 - 95)	120 (111 - 129)	99 (95 - 101)

2. Supplementary Results

2.1 Figure 1 repeated with alternative handling of concentrations below the LOQ

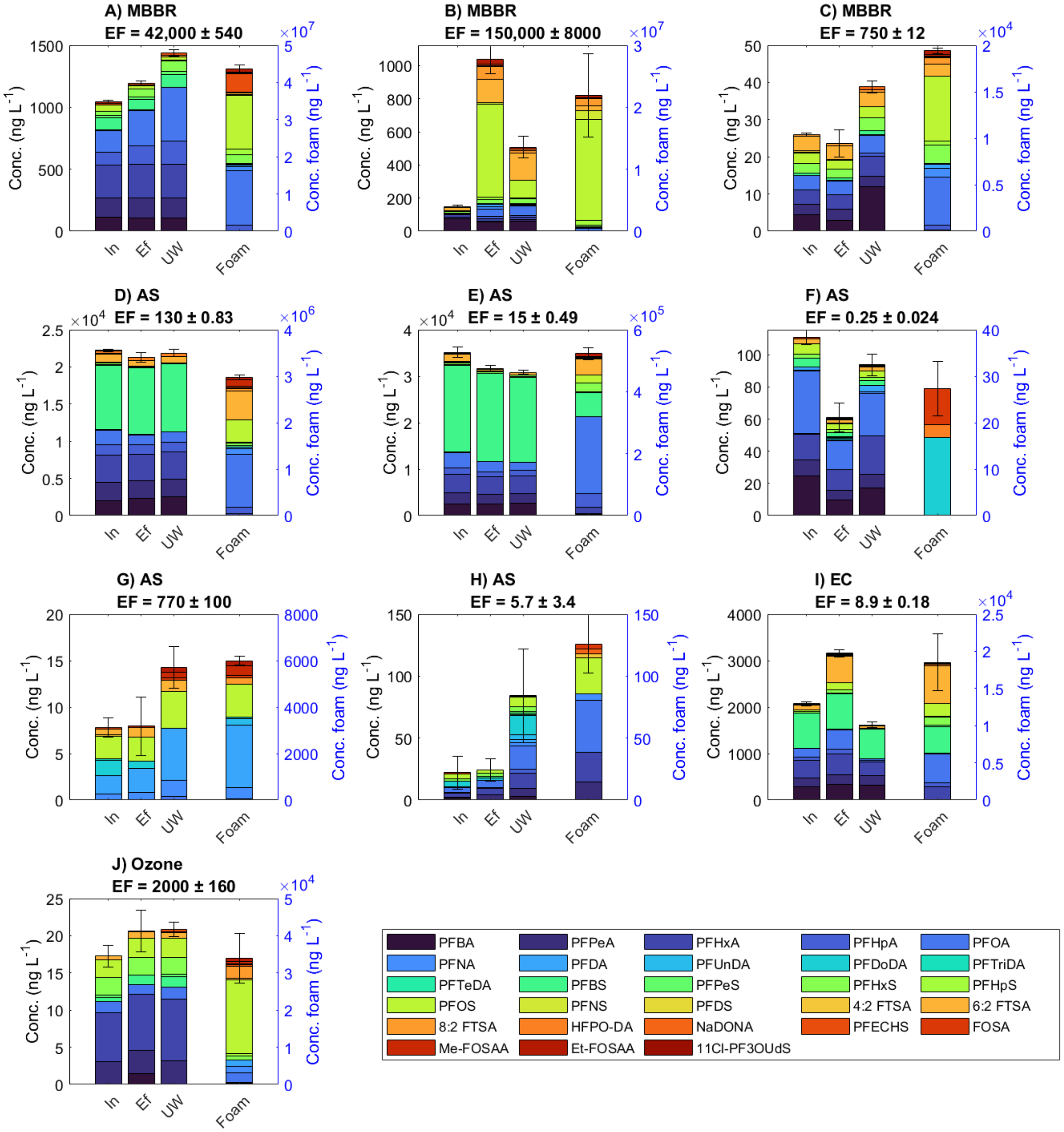

Figure SI 1: PFAS concentrations in the influent (In), effluent (Ef), water under the foam (UW) and foamate (Foam) for all treatment plants included in the study (see main text Table 1, labels of the subplots correspond to the site identifiers), with concentration below the LOQ set to zero. MBBR = moving bed biofilm reactor, AS = activated sludge, EC = electrocoagulation, Ozone = ozonation.

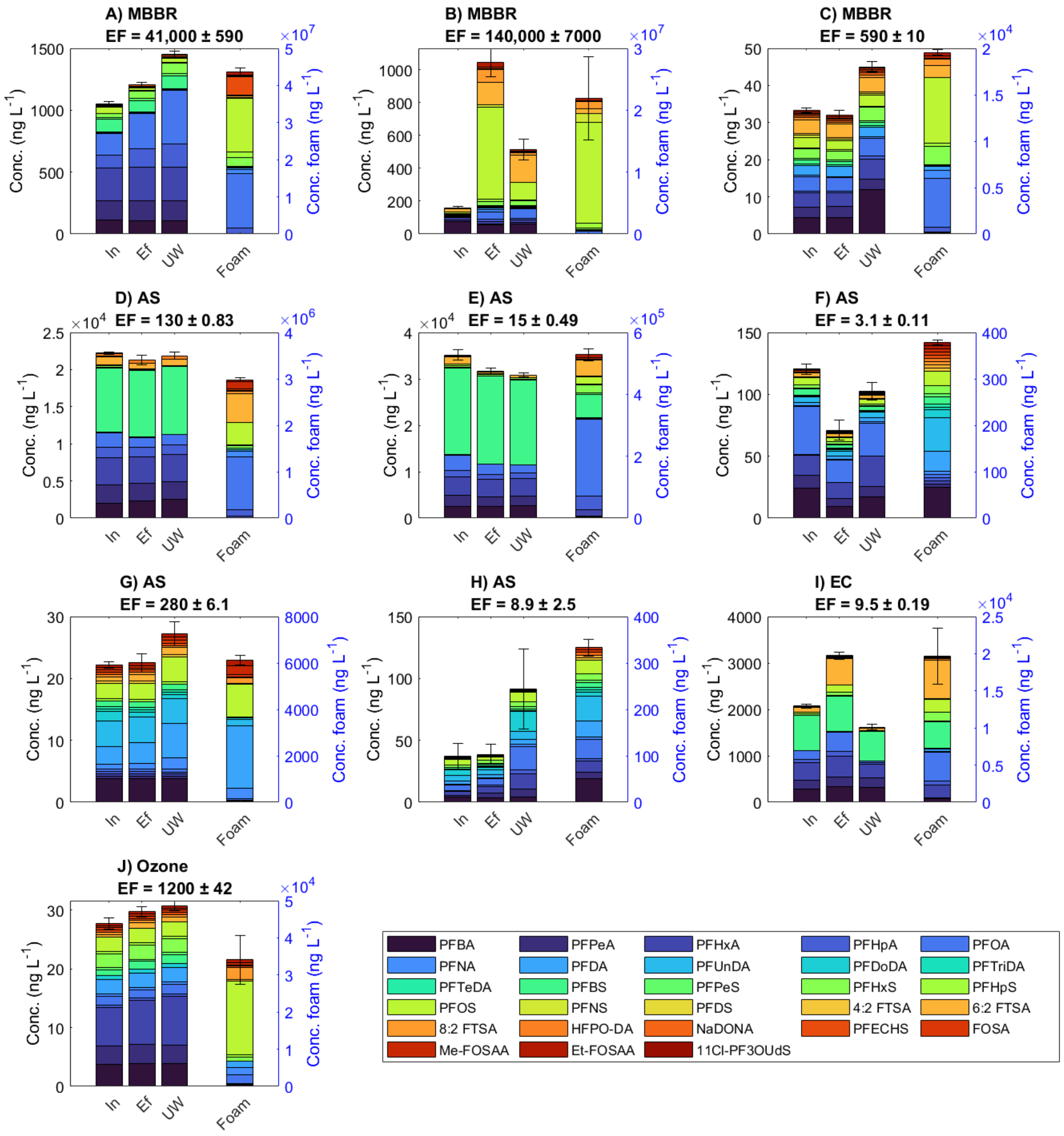

Figure SI 2: PFAS concentrations in the influent (In), effluent (Ef), water under the foam (UW) and foamate (Foam) for all treatment plants included in the study (see main text Table 1, labels of the subplots correspond to the site identifiers), with concentrations below the LOQ set to the LOQ. MBBR = moving bed biofilm reactor, AS = activated sludge, EC = electrocoagulation, Ozone = ozonation.

2.2 Mean PFAS concentrations for each site

Table SI 6: Mean PFAS concentrations (ng L⁻¹) in influent, effluent, water under the foam and foam for each site (A-J) included in the study. W When all triplicates had concentrations below the LOQ, the concentration is reported as < LOQ. When at least one triplicate had a concentration above the LOQ, the other triplicates' concentrations were set to half the LOQ, and the average of the three values was reported.

Conc. (ng L^{-1})	A				B				C			
	In	Ef	UW	Foam	In	Ef	UW	Foam	In	Ef	UW	Foam
**PFBA**	110	110	100	<2000	77	56	63	<2000	4.5	3.7	12	<100
**PFPeA**	160	160	170	380	6.7	6.3	5.5	<200	2.8	3	2.8	<10
**PFBS**	98	88	100	11000	2.1	2.1	2.5	<440	0.83	0.74	1	<22
**PFHxA**	260	270	270	180000	15	15	16	<200	3.9	3.7	5.2	130
**4:2 FTSA**	0.64	<0.40	0.3	<200	<0.40	<0.40	<0.40	<200	0.38	0.38	<0.38	11
**HFPO-DA**	<0.42	<0.40	<0.42	1500	3	5.2	8.5	<200	<0.40	<0.40	<0.40	<10
**PFPeS**	14	19	22	220000	2	2.9	3.9	240	<0.39	<0.40	<0.38	92
**PFHpA**	100	150	190	1400000	6	12	10	<200	<0.39	<0.40	1	500
**NaDONA**	<0.42	<0.40	<0.42	780	<0.40	<0.40	<0.40	<200	<0.39	<0.40	0.83	12
**PFHxS**	33	62	85	2500000	5.4	26	29	170000	2.5	2.4	3.5	2000
**PFOA**	180	290	430	15000000	2.4	46	60	400000	3.9	3.6	4.7	5200
**6:2 FTSA**	5.4	6.8	9	550000	21	140	160	720000	3.8	3.6	3.8	1300
**PFHpS**	3	3.2	5.3	1500000	<0.39	13	4.1	750000	<0.39	<0.40	<0.38	400
**PFECHS**	7.9	12	19	5000000	<0.39	0.62	0.3	32000	<0.39	<0.40	<0.38	120
**PFNA**	3.2	2.4	3.5	1100000	<0.39	15	3.6	230000	<0.39	<0.40	<0.38	890
**FOSA**	1.5	0.59	0.88	220000	<0.39	9.7	3.3	160000	<0.39	<0.40	<0.38	21
**PFOS**	48	24	30	14000000	7.5	560	110	16000000	2.8	2.3	3	7000
**PFDA**	1.9	<2.70	<2.80	470000	<2.60	10	2.9	120000	<2.60	<2.70	<2.60	480
**8:2 FTSA**	<0.42	0.37	<0.42	69000	0.31	74	15	1100000	0.56	0.51	0.64	640
**9Cl-PF3ONS**	<0.59	<0.59	<0.59	<300	<0.59	<0.59	<0.59	<300	<0.59	<0.59	<0.59	<15
**PFNS**	<0.42	<0.40	<0.42	150000	<0.39	11	2.3	1500000	0.42	<0.40	<0.38	21
**PFUnDA**	<4.50	<4.30	<4.50	21000	<4.10	<4.10	<4.30	9500	<4.30	<4.30	<4.30	<110
**Me-FOSAA**	2.5	<0.40	0.32	220000	<0.39	<0.38	<0.40	5200	<0.39	<0.40	<0.38	240
**Et-FOSAA**	9.4	1.5	1.9	780000	0.27	28	10	380000	<0.39	<0.40	<0.38	360
**PFDS**	<0.42	<0.40	<0.42	29000	<0.39	<0.38	<0.40	7300	<0.40	<0.40	<0.40	<10
**PFDoDA**	1.3	<0.73	0.7	3900	<0.70	<0.70	0.58	790	<0.72	<0.72	<0.72	<18
**11Cl-PF3OUdS**	<0.40	<0.40	<0.40	<200	<0.40	<0.40	<0.40	<200	<0.40	<0.40	<0.40	<10
**PFTriDA**	<0.42	<0.40	<0.42	230	0.28	0.27	0.47	<200	<0.40	<0.40	<0.40	<10
**PFTeDA**	<0.42	<0.40	<0.42	200	<0.39	0.53	0.94	1900	<0.39	0.43	0.26	<10

Table SI 6 continued

Conc. (ng L⁻¹)	D				E				F			
	In	Ef	UW	Foam	In	Ef	UW	Foam	In	Ef	UW	Foam
**PFBA**	2000	2300	2600	3600	2500	2600	2600	3800	24	9.5	17	<67
**PFPeA**	2500	2300	2400	4100	2400	1900	2100	2800	10	6.2	8.5	<6.7
**PFBS**	8700	8900	9100	39000	19000	19000	18000	76000	5.4	2.8	3.1	<15
**PFHxA**	3700	3600	3700	44000	4100	3800	3900	21000	16	13	24	<6.7
**4:2 FTSA**	12	15	14	<200	22	6.3	5.5	<200	<0.39	0.68	<0.40	<6.7
**HFPO-DA**	350	430	450	12000	92	85	100	1100	<0.39	0.35	<0.40	5.1
**PFPeS**	45	47	45	2500	140	120	120	4000	<0.39	<0.38	<0.40	<6.7
**PFHpA**	1300	1200	1300	130000	1300	1100	1100	45000	0.47	<0.38	<0.40	<6.7
**NaDONA**	1.7	3	3.7	620	<0.39	1.5	1.5	<200	<0.40	<0.40	<0.40	<6.7
**PFHxS**	230	110	100	44000	300	290	270	27000	2.4	1.8	1.7	<17
**PFOA**	2000	1400	1400	1100000	3400	2200	1900	250000	39	18	26	<6.7
**6:2 FTSA**	1100	780	820	620000	1700	470	430	53000	2.9	2.2	2.5	<6.9
**PFHpS**	5.3	5.5	4.9	22000	20	13	8.8	2000	<0.40	<0.40	<0.40	<6.7
**PFECHS**	2.9	3.3	3.3	5900	9.2	7.4	5.4	850	0.36	<0.38	<0.40	<6.7
**PFNA**	44	21	18	120000	42	16	11	1900	0.69	0.69	1.1	<6.7
**FOSA**	1.9	1.4	1	34000	5.8	2.4	1.9	1400	0.77	0.53	0.54	7.7
**PFOS**	89	37	30	490000	300	73	57	24000	6.4	3.3	4	<31
**PFDA**	11	1.7	<2.50	36000	16	2.3	<2.50	<1300	2.4	<2.50	3.8	<45
**8:2 FTSA**	18	2.6	3.4	44000	18	<0.37	<0.37	<200	<0.39	0.46	0.29	<6.7
**9Cl-PF3ONS**	<0.59	<0.59	<0.59	<300	<0.59	<0.59	<0.59	<300	<0.59	<0.59	<0.59	<9.8
**PFNS**	<0.40	<0.40	<0.40	<200	<0.39	<0.37	<0.37	180	<0.40	<0.40	<0.40	<6.7
**PFUnDA**	<4.00	<3.90	<4.00	<2100	<4.20	<4.00	<4.00	<2100	<4.10	<4.10	<4.30	<71
**Me-FOSAA**	72	6.1	4.5	150000	95	2.8	2.6	2900	<0.39	<0.38	<0.40	<6.7
**Et-FOSAA**	6.2	2.3	2.4	49000	97	9.4	12	10000	0.43	0.35	0.66	<6.7
**PFDS**	<0.40	<0.40	<0.40	<200	<0.40	<0.40	<0.40	<200	<0.40	<0.40	<0.40	<6.7
**PFDoDA**	<0.72	<0.72	<0.72	<360	1.2	<0.67	<0.68	<360	<0.70	0.76	<0.72	17
**11Cl-PF3OUdS**	<0.40	<0.40	<0.40	<200	<0.40	<0.40	<0.40	<200	<0.39	<0.38	0.28	<6.7
**PFTriDA**	<0.40	<0.40	<0.40	<200	0.27	<0.37	<0.37	<200	<0.39	0.69	<0.40	<6.7
**PFTeDA**	<0.38	0.39	0.55	<200	0.46	<0.37	<0.37	<200	<0.39	0.58	<0.40	<6.7

Table SI 6 continued

Conc. (ng L^{-1})	G				H				I			
	In	Ef	UW	Foam	In	Ef	UW	Foam	In	Ef	UW	Foam
**PFBA**	<3.9	<3.8	<3.8	<67	2.8	<3.7	3.5	<50	280	330	320	<500
**PFPeA**	<0.39	<0.38	<0.38	<6.7	1.2	4	6.6	14	200	210	200	<50
**PFBS**	<0.86	<0.85	<0.84	<15	<0.94	1.5	1.2	<11	750	760	630	3500
**PFHxA**	<0.39	<0.38	<0.38	6.8	3	5.1	12	24	380	440	300	1700
**4:2 FTSA**	0.26	<0.38	<0.38	5	<0.42	<0.37	0.27	5	0.67	0.75	0.59	<50
**HFPO-DA**	<0.40	<0.40	<0.40	<6.7	<0.40	<0.40	<0.40	<5.0	23	24	14	160
**PFPeS**	<0.40	<0.40	<0.40	<6.7	<0.42	<0.37	1.5	<5.0	6.2	5.3	2.8	170
**PFHpA**	<0.39	<0.38	<0.38	<6.7	0.69	0.75	3.6	<5.0	77	110	30	540
**NaDONA**	<0.40	<0.40	<0.40	<6.7	<0.42	<0.37	<0.38	4.6	<0.40	<0.40	<0.40	<50
**PFHxS**	<1.0	<1.0	<1.0	<17	1.9	2.4	4	<13	40	70	11	1100
**PFOA**	<0.39	<0.38	0.42	60	4.2	5.6	19	42	180	400	43	4000
**6:2 FTSA**	0.67	1	1.2	20	<0.44	<0.39	0.64	<5.2	110	580	53	5200
**PFHpS**	<0.39	<0.38	<0.38	6.9	<0.40	<0.40	<0.40	<5.0	2.7	8	0.54	53
**PFECHS**	<0.39	<0.38	<0.38	12	<0.40	<0.40	<0.40	<5.0	2.3	4.8	0.74	89
**PFNA**	0.69	0.84	1.7	470	0.92	0.57	2.2	5.4	2.4	13	0.27	51
**FOSA**	0.27	<0.38	0.37	100	<0.42	<0.37	<0.38	4.7	1.1	5.4	0.61	87
**PFOS**	2.4	2.6	4	1400	4.3	3	7.9	29	24	150	11	1700
**PFDA**	2.4	3	5.6	2700	<2.8	<2.5	3.4	<34	3	11	<2.7	<340
**8:2 FTSA**	<0.39	<0.38	<0.38	260	<0.40	<0.40	<0.40	<5.0	<0.39	5.8	<0.40	<50
**9Cl-PF3ONS**	<0.59	<0.59	<0.59	<9.8	<0.59	<0.59	<0.59	<7.4	<0.59	<0.59	<0.59	<74
**PFNS**	<0.40	<0.40	<0.40	<6.7	<0.40	<0.40	<0.40	<5.0	<0.40	<0.40	<0.40	<50
**PFUnDA**	<4.1	<4.1	<4.0	270	<4.5	<4.0	5	<54	<4.3	<4.3	<4.3	<540
**Me-FOSAA**	<0.39	<0.38	0.59	410	0.88	<0.37	<0.38	<5.0	1.1	5.3	0.29	40
**Et-FOSAA**	<0.39	0.27	0.5	220	<0.40	<0.40	<0.40	<5.0	1.3	13	0.81	75
**PFDS**	<0.40	<0.40	<0.40	<6.7	<0.40	<0.40	<0.40	<5.0	<0.40	<0.40	<0.40	<50
**PFDoDA**	1.6	0.83	<0.68	73	4.2	1.9	16	<9.1	1.4	1.4	1.2	91
**11Cl-PF3OUdS**	<0.40	<0.40	<0.40	<6.7	<0.40	<0.40	<0.40	<5.0	<0.40	<0.40	<0.40	<50
**PFTriDA**	0.29	<0.38	<0.38	<6.7	<0.42	<0.37	0.72	<5.0	<0.40	<0.40	<0.40	<50
**PFTeDA**	<0.39	<0.38	<0.38	4.7	<0.42	<0.37	0.27	<5.0	<0.40	<0.40	<0.40	<50

Table SI 6 continued

Conc. (ng L^{-1})	J			
	In	Ef	UW	Foam
**PFBA**	<3.8	2.7	<3.8	160
**PFPeA**	3.1	3.2	3.2	100
**PFBS**	0.71	1.3	1.4	31
**PFHxA**	6.5	7.5	8.3	230
**4:2 FTSA**	<0.40	<0.40	<0.40	<20
**HFPO-DA**	<0.40	<0.40	<0.40	<20
**PFPeS**	0.34	<0.38	0.33	<20
**PFHpA**	<0.38	<0.38	<0.38	93
**NaDONA**	<0.40	<0.40	<0.40	<20
**PFHxS**	2.4	2.4	2.3	1100
**PFOA**	1.5	1.4	1.6	2500
**6:2 FTSA**	0.47	0.87	0.78	490
**PFHpS**	<0.38	<0.38	<0.38	640
**PFECHS**	<0.38	<0.38	0.32	320
**PFNA**	<0.38	<0.38	<0.38	1800
**FOSA**	<0.38	<0.38	<0.38	260
**PFOS**	2.4	2.5	2.5	20000
**PFDA**	<2.5	<2.5	<2.6	1700
**8:2 FTSA**	<0.38	0.26	0.29	3200
**9Cl-PF3ONS**	<0.59	<0.59	<0.59	<30
**PFNS**	<0.40	<0.40	<0.40	<20
**PFUnDA**	<4.3	<4.3	<4.3	<210
**Me-FOSAA**	<0.38	<0.38	<0.38	670
**Et-FOSAA**	<0.38	<0.38	<0.38	820
**PFDS**	<0.40	<0.40	<0.40	<20
**PFDoDA**	<0.69	<0.69	<0.69	35
**11Cl-PF3OUdS**	<0.40	<0.40	<0.40	<20
**PFTriDA**	<0.40	<0.40	<0.40	<20
**PFTeDA**	<0.40	<0.40	<0.40	<20

2.3 Concentrations after the TOP assay

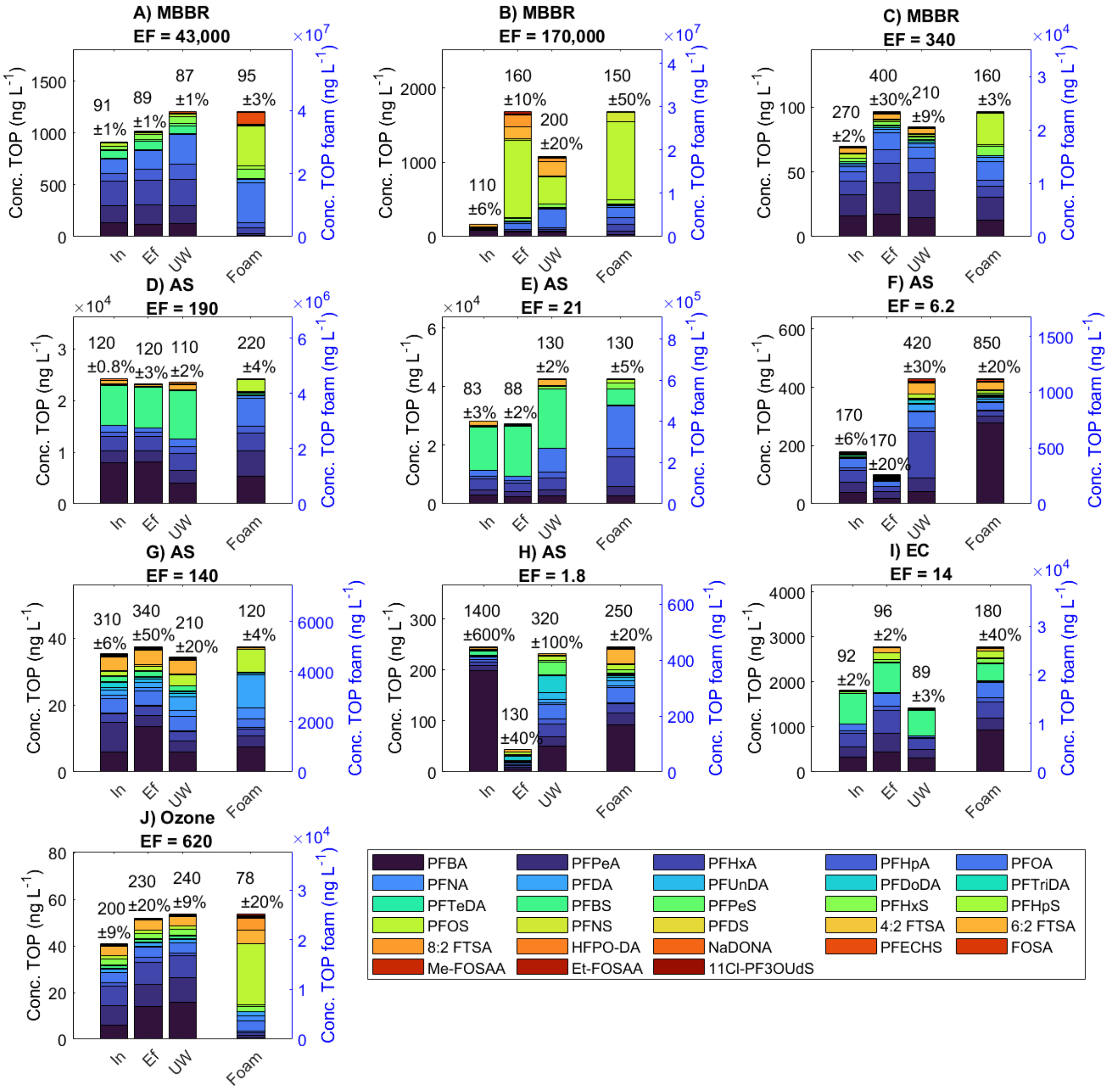

Figure SI 3: PFAS concentrations after the TOP assay in the influent (In), effluent (Ef), water under the foam (UW) and foamate (Foam) for all treatment plants included in the study (see main text Table 1, labels of the subplots correspond to the site identifiers). Concentrations below the LOQ were set to 0.5·LOQ. Foamate concentrations are presented on the y-axis on the right. Titles give the enrichment factors (EF) calculated based on the concentrations after TOP. The text above the bars gives the molar percentage of PFAS compared to the target measurement, i.e. percentages above 100 % indicate an increased ΣPFAS concentration due to precursor degradation. Concentrations below 100 % are probably due to measurement uncertainties. The standard deviations are based only on the variability in target concentrations (n = 3), since TOP assays were done without replicates. MBBR = moving bed biofilm reactor, AS = activated sludge, EC = electrocoagulation, Ozone = ozonation.

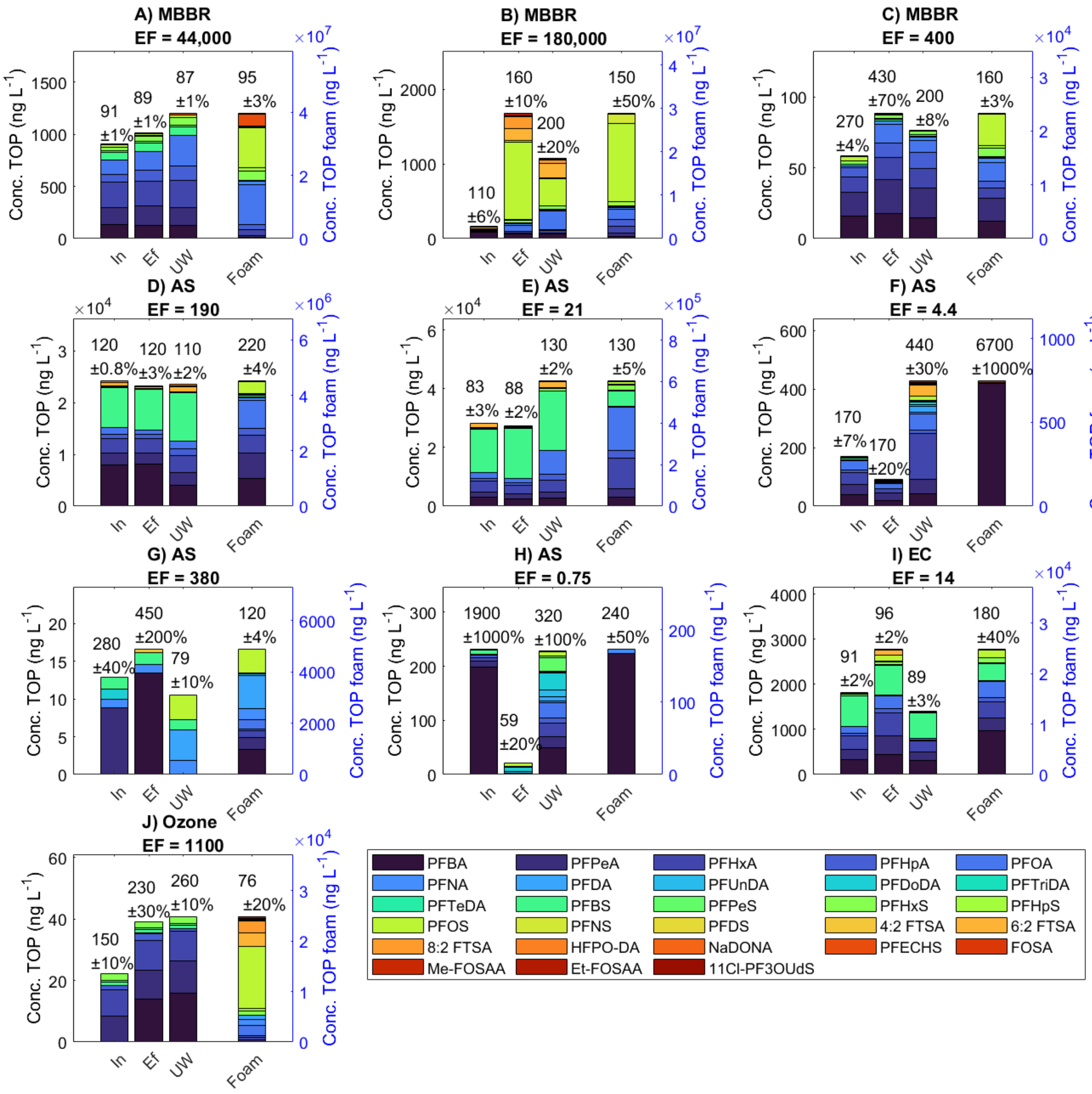

Figure SI 4: PFAS concentrations after the TOP assay in the influent (In), effluent (Ef), water under the foam (UW) and foamate (Foam) for all treatment plants included in the study (see main text Table 1, labels of the subplots correspond to the site identifiers), with concentrations below the LOQ set to zero. MBBR = moving bed biofilm reactor, AS = activated sludge, EC = electrocoagulation, Ozone = ozonation.

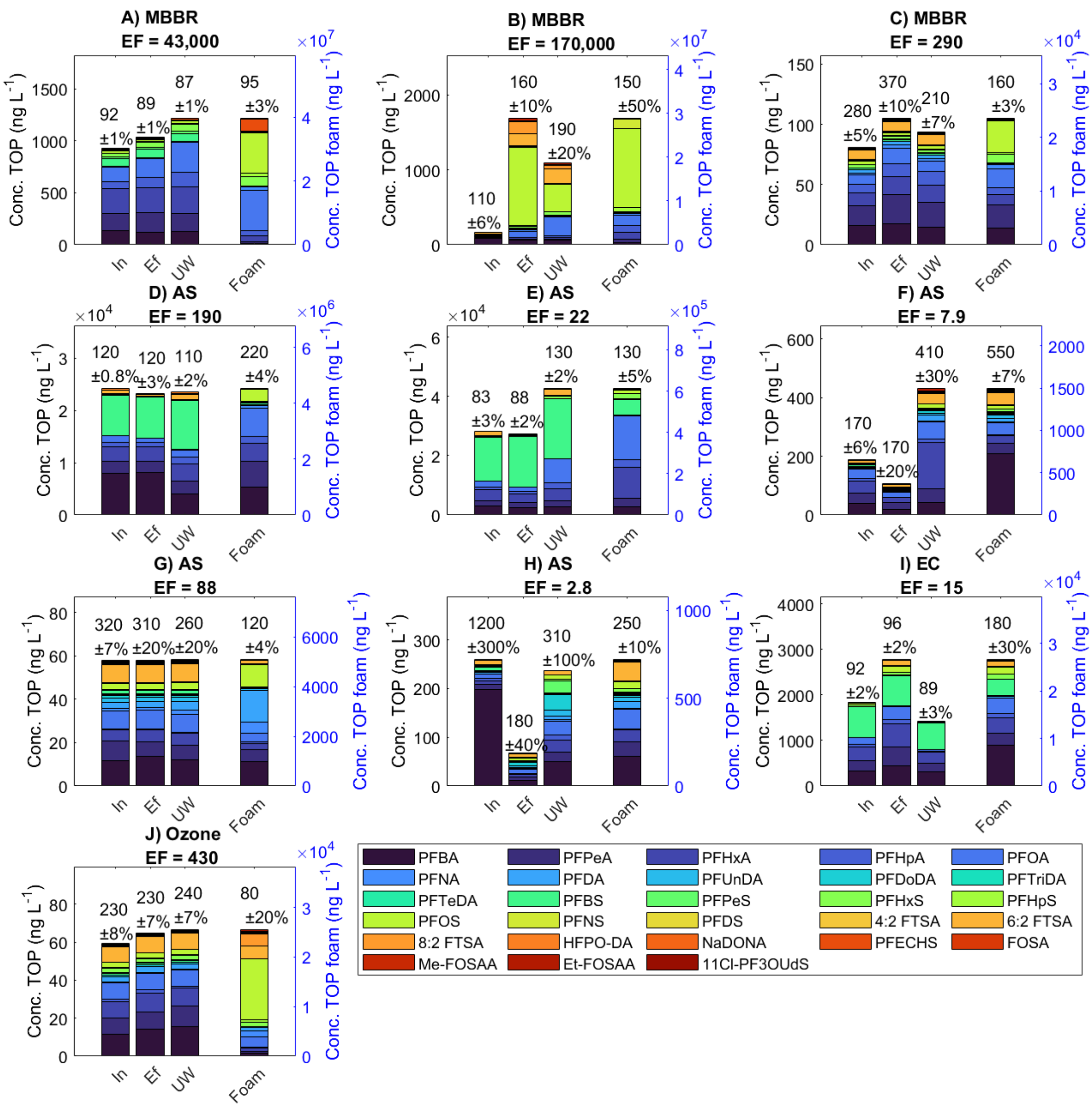

Figure SI 5: PFAS concentrations after the TOP assay in the influent (In), effluent (Ef), water under the foam (UW) and foamate (Foam) for all treatment plants included in the study (see main text Table 1, labels of the subplots correspond to the site identifiers), with concentrations below the LOQ set to the LOQ. MBBR = moving bed biofilm reactor, AS = activated sludge, EC = electrocoagulation, Ozone = ozonation.

2.4 Quantification of required foam fraction for increased long-chain PFAA removal

When ignoring sorption to sludge and reactive transformation of precursors into target PFAS, the mass balance over a foam-forming process can be written as follows, provided that the foam would be removed from the top of the reactor:

$$\frac{dVC_{bulk}}{dt} = \varphi_{In}C_{In} - \varphi_{Ef}C_{Ef} - \varphi_{Foam}C_{Foam}$$

Here, V is the volume of the reactor (m^3), C_{bulk}, C_{In}, C_{Ef} and C_{Foam} the PFAS concentration in the reactor, influent, effluent and foam, respectively (all mol m^{-3}), and φ_{In}, φ_{Ef} and φ_{Foam} the influent, effluent and foam flow rate (all $m^3\,hr^{-1}$), respectively. At steady state and constant reactor volume, this means that:

$$\varphi_{In}C_{In} - \varphi_{Ef}C_{Ef} - \varphi_{Foam}C_{Foam} = 0\text{, and:}$$

$$\varphi_{In} = \varphi_{Ef} + \varphi_{Foam} \rightarrow \ \varphi_{Ef} = \ \varphi_{In} - \varphi_{Foam}$$

Then:

$$\varphi_{In}C_{In} = (\varphi_{In} - \varphi_{Foam})C_{Ef} + \varphi_{Foam}C_{Foam}$$

$$C_{In} = (1 - \frac{\varphi_{Foam}}{\varphi_{In}})C_{Ef} + \frac{\varphi_{Foam}}{\varphi_{In}}C_{Foam}$$

$$C_{In} - C_{Ef} = \frac{\varphi_{Foam}}{\varphi_{In}}(C_{Foam} - C_{Ef})$$

$$\frac{\varphi_{Foam}}{\varphi_{In}} = \frac{C_{In} - C_{Ef}}{C_{Foam} - C_{Ef}}$$

At a removal of RE %:

$$C_{Ef} = \left(1 - \frac{RE}{100}\right)C_{In}$$

Then:

$$\frac{\varphi_{Foam}}{\varphi_{In}} = \frac{C_{In} - \left(1 - \frac{RE}{100}\right)C_{In}}{C_{Foam} - \left(1 - \frac{RE}{100}\right)C_{In}} \quad \text{(Foam Fraction)}$$

The next step is to relate the foam concentration to the effluent concentration. Using the analysis by Stevenson and Li (2017)[1], C_{Foam} can be related to C_{Ef} as below, with Γ the surface excess concentration of PFAS (mol m^{-2}) and r_{32} the Sauter mean bubble radius of the foam (m):

$$C_{Foam} = \ C_{Ef} + \frac{3\Gamma}{r_{32}}$$

The equilibrium relation between the surface excess and the effluent concentration is given by an adsorption isotherm. For simplicity, a Henry's law isotherm is used, which is relatively realistic at low concentrations, with Henry's constant K_H (m):

$$\Gamma = \ K_H \cdot C_{Ef}$$

Combining the two equations above and introducing the variable $K = \left(1 + \frac{3K_H}{r_{32}}\right)$:

$$C_{Foam} = \ C_{Ef} + \frac{3K_H \cdot C_{Ef}}{r_{32}} = \ \left(1 + \frac{3K_H}{r_{32}}\right)C_{Ef} = K \cdot C_{Ef} = K \cdot \left(1 - \frac{RE}{100}\right)C_{In}$$

Using the above definition of C_{Foam} in the equation for the foam fraction:

$$\frac{\varphi_{Foam}}{\varphi_{In}} = \frac{C_{In} - \left(1 - \frac{RE}{100}\right) C_{In}}{K \cdot \left(1 - \frac{RE}{100}\right) \cdot C_{In} - \left(1 - \frac{RE}{100}\right) C_{In}}$$

$$\frac{\varphi_{Foam}}{\varphi_{In}} = \frac{1 - \left(1 - \frac{RE}{100}\right)}{K \cdot \left(1 - \frac{RE}{100}\right) - \left(1 - \frac{RE}{100}\right)}$$

$$\frac{\varphi_{Foam}}{\varphi_{In}} = \frac{\frac{RE}{100}}{\left(1 - \frac{RE}{100}\right) \cdot (K - 1)}$$ (Equation S1, Foam Fraction)

There are weaknesses in this analysis that must be pointed out. As aforementioned, the analysis ignores adsorption to sludge and reactive transformation of precursors. Secondly, a Henry adsorption isotherm is only realistic at low concentrations and frequently the more accurate, but more complex, Langmuir isotherm is used. Thirdly, in reality, r_{32} is a variable that will often change when the foam fraction increases. Changing the foam fraction without changing the bubble radius is difficult, since the wetness of the foam is a function of bubble size, so K is only an independent constant if the foam fraction is increased without increasing the foam wetness. Finally, as pointed out in the main text, the foam is not removed from the water surface in any of the plants under investigation in this study, and the retention time of the foam was likely higher than that of the water in most plants.

Despite these limitation, Equation S1 may be used to roughly estimate the required foam fraction that would be necessary to achieve certain levels of long-chain PFAA removal. For this calculation, the removal and enrichment of Σlong-chain PFAA were used, since these compounds are removable with foam fractionation, and using summed concentrations moderates the effects of non-detect concentrations. As visible from Figure SI 6b, at sites D and E, an approximately seven-fold increase in volumetric foam formation may already result in a Σlong-chain PFAA removal of 80 %. Since the calculated foam fractions at these two sites were currently both < 0.5 % (Figure SI 6a), this may be achievable. Furthermore, at site J, a removal of > 99 % would require a foam fraction of only 3 % (Figure SI 6a). However, it should be stressed that these calculations are approximations only and that artificially increasing the foam formation while keeping the bubble size (and thus foam wetness and relative surface area) constant may not be possible.

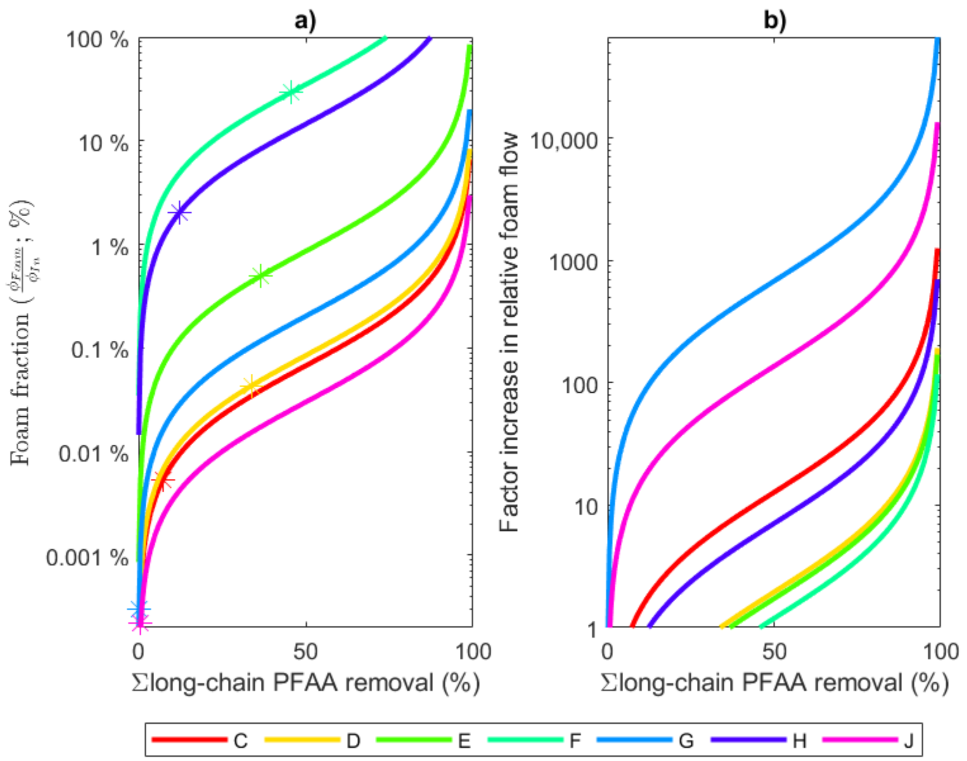

Figure SI 6: Foam fraction (a) and increase in foam fraction (b) required to reach a certain Σlong-chain PFAA removal. Asterisks in a) represent the Σlong-chain PFAA removal and calculated foam fraction as found from the concentrations obtained in this study, i.e. during normal plant operation. Only sites for which the measured Σlong-chain PFAA removal was positive were included, since for the remaining sites the calculated foam fractions would be negative. The letters in the legend correspond to the site identifiers given in main text Table 1. These plots are rough approximations only, since the calculations ignore sorption to sludge and reactive transformation of PFAS and assume the relative surface area of the foam to be constant independent of foam fraction, which is not realistic.

2.4 Figure 3 repeated with alternative handling of concentrations below the LOQ

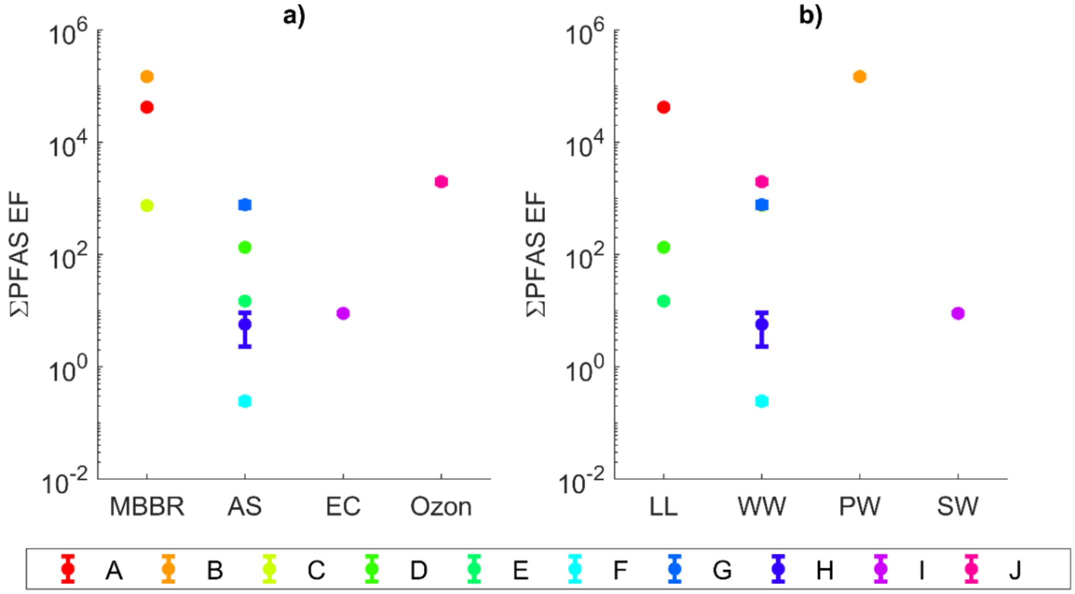

Figure SI 7: ΣPFAS EF grouped by a) treatment process and b) water type, with concentrations below the LOQ set to zero. MBBR = moving bed biofilm reactor, AS = activated sludge, EC = electrocoagulation, Ozon = ozonation, LL = landfill leachate, WW = wastewater, PW = process water, SW = stormwater runoff from landfill bottom ash collection site. Error bars represent the standard deviation (sd) within the EF for each plant (n = 3 for foamate as well as influent concentrations), but are difficult to see for all plants except H, because the sd was relatively small. The letters in the legend correspond to the site identifiers given in main text Table 1.

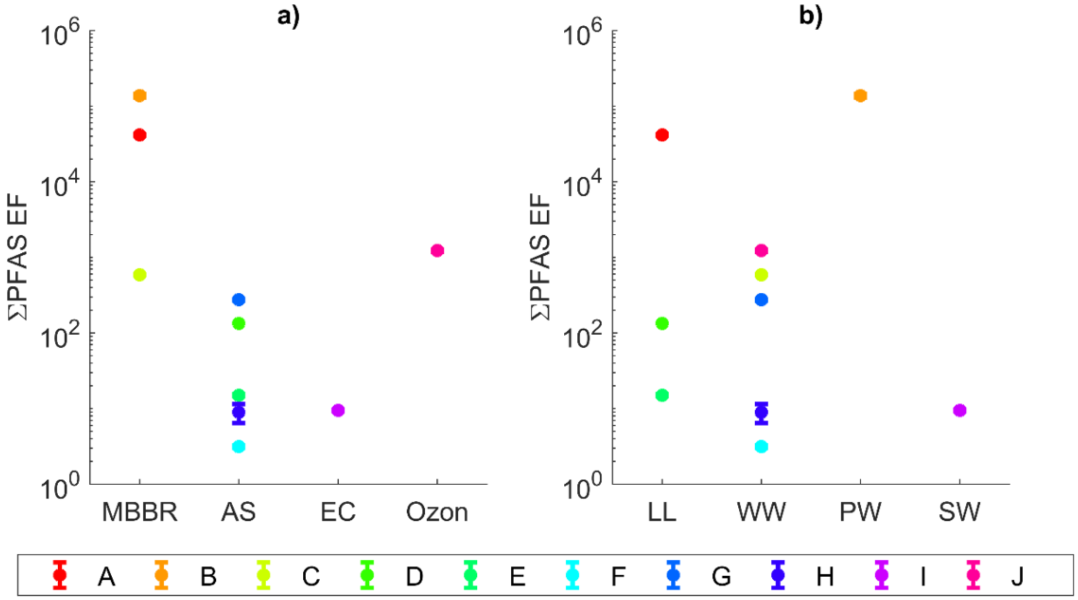

Figure SI 8: ΣPFAS EF grouped by a) treatment process and b) water type, with concentrations below the LOQ set to the LOQ. MBBR = moving bed biofilm reactor, AS = activated sludge, EC = electrocoagulation, Ozon = ozonation, LL = landfill leachate, WW = wastewater, PW = process water, SW = stormwater runoff from landfill bottom ash collection site. Error bars represent the standard deviation (sd) within the EF for each plant (n = 3 for foamate as well as influent concentrations), but are difficult to see for all plants except H, because the sd was relatively small. The letters in the legend correspond to the site identifiers given in main text Table 1.

2.5 General chemistry results

Table SI 7: General chemistry of the influent for all sites.

	A	B	C	D	E	F	G	H	I	J
**Aluminum (µg L^{-1})**	130	140	1500	67	250	420	310	1300	2000	55
**Arsenic (µg L^{-1})**	3	<0.5	1	40	110	2.4	5.5	<0.5	5.7	<0.5
**Barium (µg L^{-1})**	500	3.1	9.0	360	440	31	45	71	100	7.4
**Calcium (mg L^{-1})**	130	20	44	240	160	76	140	95	190	39
**Cadmium (µg L^{-1})**	0.15	0.07	<0.05	0.76	1.2	0.14	0.09	0.55	0.98	<0.05
**Cobalt (µg L^{-1})**	2.3	6.5	0.34	8.9	33	0.64	0.77	0.64	4	1.4
**Chromium (µg L^{-1})**	1.6	8.4	<0.9	45	350	2.3	3.8	120	7.8	<0.9
**Copper (µg L^{-1})**	18	43	16	3.3	210	52	17	42	480	5.1
**Iron (mg L^{-1})**	5.8	29	0.19	0.98	5.6	5.6	2.4	0.85	0.91	0.48
**Mercury (µg L^{-1})**	<0.02	<0.02	<0.02	<0.02	0.03	0.03	0.02	0.07	0.03	<0.02
**Potassium (mg L^{-1})**	180	14	21	460	940	25	26	42	450	21
**Magnesium (mg L^{-1})**	49	2.6	9.0	200	140	9.3	58	3.1	21	6.3
**Manganese (µg L^{-1})**	380	150	43	1100	760	95	57	35	160	91
**Molybdenum (µg L^{-1})**	7.4	4.8	2.9	480	27	2.8	1.5	180	140	1.9
**Sodium (mg L^{-1})**	550	2700	64	1500	6400	110	340	62	1700	74
**Nickel (µg L^{-1})**	18	21	2.7	140	410	6.3	4.3	3.1	53	2.6
**Lead (µg L^{-1})**	1.5	2	<0.5	1.1	8.7	11	5.8	41	33	<0.5
**Vanadium (µg L^{-1})**	1.4	4.4	0.29	39	180	2.1	1.3	3.7	8.5	<0.2
**Zinc (µg L^{-1})**	25	93	17	42	230	100	80	100	130	20
**Chloride (mg L^{-1})**	650	1600	100	2300	13000	150	<1.0	67	2700	94
**Fluoride (mg L^{-1})**	0.24	<0.4	0.44	0.96	4.7	0.27	0.23	1.1	<0.4	0.22
**Total phosphorus**	0.98	21	1.7	6.7	44	5.1	5.6	7.2	0.21	0.5
**Turbidity (FNU)**	140	400	14	280	29	100	37	72	270	3.4
**Conductivity (mS m^{-1})**	360	1100	98	1300	3300	130	300	77	980	72
**pH**	7.7	8.1	7.7	7.4	7.4	8	7.5	8.5	7.9	7
**Total nitrogen (mg L^{-1})**	58	85	51	420	1200	55	45	24	18	20
**Total organic carbon (TOC, mg L^{-1})**	41	1000	30	230	600	120	37	46	85	14

Bibliography

(1) Stevenson, P.; Li, X. *Foam Fractionation - Principles and Process Design*, 1st ed.; Taylor & Francis: Boca Raton, 2014.
